# The selenium-enriched *Rhodotorula mucilaginosa* JAASRY1 improved oxidative stress during the aging process via the gut-liver-brain axis

**DOI:** 10.3389/fmicb.2026.1809542

**Published:** 2026-06-02

**Authors:** Mubai Sun, Da Li, Luyao Wang, Yuguang He, Yue Hu, Min Yang, Zhengyang Luo, Xinyu Miao, Sheng Yu, Mei Hua, Honghong Niu, Jinghui Wang

**Affiliations:** 1 Institute of Agro-food Technology, Jilin Academy of Agricultural Sciences (Northeast Agricultural Research Center of China), Changchun, China; 2 Jilin Agricultural University, Changchun, China; 3 Agronomy of Food Science and Technology, Yanbian University, Yanbian, China; 4 Changchun Bioxun Biotech Co., Ltd, Changchun, China

**Keywords:** aging, brain-gut axis, gut microbiota, liver-gut axis, oxidative stress, selenium-enriched *Rhodotorula mucilaginosa*

## Abstract

**Background:**

Oxidative stress, induced by the aging process, has been demonstrated to engender a variety of deleterious effects on the organism. The effectiveness of selenium-enriched *Rhodotorula mucilaginosa* remains unproven.

**Methods:**

In this study, selenium-enriched *Rhodotorula mucilaginosa JAASRY1* (*Se-RMSRY*) was comprehensively characterized. Scanning electron microscopy coupled with energy-dispersive X-ray spectroscopy was employed to analyze its morphology and the elemental distribution of selenium. Furthermore, the specific chemical forms of biogenic selenium in *Se-RMSRY* were identified and quantified using high-performance liquid chromatography-inductively coupled plasma mass spectrometry. The potential of *Se-RMSRY* to exert antioxidant mechanisms for anti-aging effects was then systematically elucidated. Animal behavioral tests demonstrated behavioral differences induced by *Se-RMSRY*, thereby evaluating the improvement in aging phenotypes. Histopathological analysis of the hippocampus, liver, and intestine was performed to examine structural integrity and pathological changes. Intracellular reactive oxygen species (ROS) levels were detected using fluorescence-based assays to confirm oxidative stress mitigation. Western blotting was employed to validate the expression of aging-related proteins. Additionally, gut microbiome analysis via 16S rRNA sequencing was carried out to explore the composition and diversity of gut microbiota, thereby uncovering potential links between antioxidant activity and gut health in the anti-aging process.

**Results:**

The results showed that selenomethionine (SeMet) was the predominant bioselenium composition of *Se-RMSRY*, with a concentration of 1213.16 mg/kg. Furthermore, *Se-RMSRY* significantly enhanced intestinal homeostasis by enriching beneficial *Lactobacillus* sp. populations and reprogramming microbial metabolism toward carbohydrate utilization while suppressing amino acid metabolism in aging mice (*p* < 0.05). Systemic antioxidant capacity was augmented via coordinated activation of the Nrf2/NQO1/HO-1 signaling pathway and glutathione metabolism, with concomitant reductions in hepatic and intestinal oxidative stress markers (*p* < 0.05). The intervention attenuated apoptosis through Bcl-2/Bax pathway modulation and improved gut barrier integrity (*p* < 0.05), while hippocampal CA1 neuronal preservation correlated with reduced anxiety-like behaviors and enhanced cognitive performance (*p* < 0.05).

**Conclusion:**

These findings establish *Se-RMSRY* as a promising dietary intervention against age-related pathologies through gut–microbiota–organ axis regulation, thereby addressing the previously unproven efficacy of *Se-RMSRY* in counteracting aging-related oxidative stress.

## 1 Introduction

Aging is a complex biological process accompanied by accumulation of DNA damage, mitochondrial dysfunction, loss of protein homeostasis, and metabolic imbalance, leading to tissue and organ dysfunction, which in turn triggers the development of chronic degenerative diseases (Ferrucci et al., 2020; Militello et al., 2024). The World Health Organization (WHO) predicts that the number of elderly people will increase exponentially by 2050, suggesting that exploring strategies to either slow down the ageing process or reduce its associated metabolic demands represents a particularly salient research priority (Rudnicka et al., 2020). It has been hypothesized that disruption of the oxidative defenses, driven by excessive accumulation of reactive oxygen species (ROS), constitutes a pivotal factor in triggering the aging process. Although endogenous antioxidant enzymes, including superoxide dismutase (SOD) and glutathione peroxidase (GSH-Px), can neutralize ROS, their enzymatic activity and expression levels progressively decline during aging, concomitant with the deterioration of overall antioxidant defense systems (Kim et al., 2021). Notably, the aging process is pathologically linked to the sustained presence of chronic low-grade inflammation, which reciprocally exacerbates ROS generation through redox-sensitive signaling pathways (Schaum et al., 2020). This phenomenon, known as cross-fertilization of inflammation and oxidative stress, has been identified as a significant contributor to the onset of organ damage (Schaum et al., 2020). The strategic administration of bioactive dietary components to achieve homeostatic modulation of redox equilibrium represents a critical therapeutic strategy, particularly when implemented through chronotherapeutic approaches targeting age-related oxidative perturbations (Wang et al., 2025). Certain plant-derived bioactive compounds have been shown to alleviate oxidative stress and modulate immune function through their influence on the gut microbiota (Chen et al., 2023; Hassan et al., 2023; Su et al., 2023).

Selenium is an essential component of antioxidant enzymes that regulate cellular redox status by scavenging free radicals in various biological processes, particularly redox reactions (Ikram et al., 2021; Rua et al., 2023; Staneviciene et al., 2022). Recent advances have enabled the modulation of diverse physiological activities in organisms through selenium supplementation in plant, animal, or microbial sources (Che et al., 2025; D'Amato et al., 2020; Gao et al., 2024; Newman et al., 2019; Wang et al., 2024). Yeasts are characterized by versatile metabolic capabilities, endowing them with promising application prospects in biotransformation (Huang et al., 2025). Selenium-enriched yeast-based products have attracted considerable scientific interest following emerging evidence demonstrating enhanced bioavailability and therapeutic advantages of organic selenium compounds compared with their inorganic counterparts (Sumana et al., 2023). In yeast cells, the sulfur moiety in sulfur-containing amino acids is substituted by inorganic selenium and, which is subsequently assimilated into organic forms, notably SeMet (Hassan et al., 2022; M. Kieliszek and Błażejak, 2013). Accumulating evidence indicates that yeast strains such as *Saccharomyces* spp. and *Rhodotorula* spp. are capable of intracellular selenium accumulation (Chen et al., 2019; Xu et al., 2023). In contrast to conventional selenium-enriched yeast (e.g., *Saccharomyces cerevisiae*), which mainly relies on glutathione and selenoproteins for antioxidant activity, *Rhodotorula mucilaginosa* additionally synthesizes potent carotenoid antioxidants while retaining efficient selenium biotransformation capacity (Kieliszek et al., 2023). *Rhodotorula mucilaginosa* produces commercially valuable carotenoids, which are widely utilized as nutraceutical supplements (Kieliszek et al., 2023). Emerging research has increasingly investigated *Rhodotorula mucilaginosa* for its dual functional capacity to enhance nutritional profiles and modulate flavor characteristics during fermentation processes (Liang et al., 2025; Ma et al., 2025; Wang et al., 2024). Notably, cell wall components and bioactive metabolites derived from *Rhodotorula mucilaginosa* exhibit immunomodulatory capabilities that regulate key physiological functions in host organisms (Kang et al., 2023). Based on these advantages, *Rhodotorula mucilaginosa* exhibits broad application prospects in multiple fields. In traditional fermented foods such as fruit wine, it can significantly improve the flavor and antioxidant activity of products (Liu et al., 2025). In the field of functional foods, selenium-enriched *Rhodotorula mucilaginosa* can serve as an organic selenium source with high bioavailability for selenium-fortified products. Its combination of cell wall polysaccharides and carotenoids can be developed into functional foods with immune-enhancing or metabolic health-regulating effects (Díaz-Navarrete et al., 2024; Ge et al., 2021). In the pharmaceutical field, yeast polysaccharides derived from this strain show anti-inflammatory, neuroprotective and antitumor potential, and can be used as candidate raw materials for lead compounds or oral immunomodulators (Li et al., 2020; Hamidi et al., 2020; Wu et al., 2025). Collectively, selenium-enriched *Rhodotorula mucilaginosa* represents a multifunctional modulator of cellular redox homeostasis with enhanced therapeutic potential.

Accumulating evidence demonstrates that gut microbiota play pivotal roles in modulating numerous metabolic processes linked to aging. Under metabolic dysregulation, gut microbial homeostasis undergoes characteristic shifts, characterized by depletion of traditional commensal species and concurrent proliferation of pathobionts. This microbial community dynamics drives alterations in microbe-derived metabolites, exerting detrimental effects on host physiology that exacerbate metabolic dysregulation. These reciprocal interactions establish a pathological feedback loop between gut microbiota remodeling and multiorgan dysfunction during disease progression (Guo et al., 2023; Loh et al., 2024). Substantial research efforts are actively investigating targeted microbial interventions to preserve cognitive performance, optimize immune competence, and attenuate age-related organ degeneration through precision modulation of gut microbiota composition and function (Pan et al., 2022). These studies are facilitated by the complex interplay between the gut microbiome and the bidirectional communication network known as the gut-liver/gut-brain axis, where microbial metabolites (e.g., short-chain fatty acids) modulate hepatic detoxification and neuroinflammation via vagal signaling (Cryan et al., 2020; Zhang et al., 2025). Dysbiosis-driven depletion of *Bifidobacterium* sp. and *Lactobacillus* sp. exacerbates oxidative stress by reducing butyrate-mediated Nrf2 activation (Kundu et al., 2019).

The anti-aging potential of selenium-enriched *Rhodotorula mucilaginosa* in mammals remains unexplored in systematic investigations, particularly regarding its pleiotropic effects across multiple organ systems. Building upon existing evidence, this investigation pioneered a systematic exploration of the mechanistic role of selenium-enriched *Rhodotorula mucilaginosa* in attenuating age-related oxidative stress through the gut-liver axis/ gut-brain axis. We hypothesized that *Rhodotorula mucilaginosa* remodels gut microbiota to attenuate age-related oxidative damage. In addition, the study set out to compare the antioxidant effects of selenium-enriched *Rhodotorula mucilaginosa* and sodium selenite, addressing a critical knowledge gap in selenium bioavailability and its impact on cross-organ redox homeostasis.

## 2 Methods

### 2.1 Selenium-enriched *Rhodotorula mucilaginosa* preparation

The *Rhodotorula mucilaginosa JAASRY1 (RMSRY)* was isolated and identified by the Jilin Academy of Agricultural Sciences and deposited in the China General Microbiological Culture Collection Center (No. 22900). *RMSRY* was activated in LB medium and incubated in shaker at 180 r/min at 30 °C for 48 h to obtain the seed liquid (Miao et al., 2024). The activated seed solution was inoculated into LB culture medium (sodium selenite added at 20.16 mg/L) at 8% (v/v), shaking speed 180 r/min, and incubated at 30 °C for 32 h. The resulting organisms were dried at 45 °C, crushed and sieved.

### 2.2 Characterization of micromorphology and properties of *se-RMSRY*

A scanning electron microscope (SU800, Hitachi, Japan) was used to observe the micromorphology of *RMSRY*. An X-ray energy spectrometer (ULTIMMAX 100, Oxford, UK) was used to collect characteristic X-ray photons within the SEM images to observe the distribution of different elements. High Performance Liquid Chromatography (LC-20AT, Shimadzu, Japan) -Inductively Coupled Plasma Mass Spectrometry (iCAPQ, Thermo Fisher, USA) was applied to analyze the selenium morphology of *RMSRY*. Samples were processed as follows: 1 g of sample was mixed with 5 mL Tris–HCl, sonicated for 30 min and then incubated at 50 °C, 250 r/min, for 18 h. 20 mg of protease XIV was added. After continuing to incubate the sample at 37 °C for 18 h, it was centrifuged at 4 °C, 10,000 r/min for 30 min, and the supernatant was filtered through 0.22 μm aqueous filtration membrane. HPLC conditions: Anion-exchange chromatography column (250 × 4.1 mm, 10 μm, Hamilton, Switzerland) and anion-exchange chromatography guard column (25 × 2.3 mm, 25 mm, 12–20 μm, Hamilton, Switzerland) were used. The mobile phase was ultrapure water, and the elution mode was isocratic elution. The flow rate was set at 1.0 mL/min, and the injection volume was 100 μL. ICP-MS conditions: radiofrequency power of 1,550 W, nebulizer chamber temperature of 2.5 °C, cooling gas of 14 L/min, carrier gas flow rate of 0.50 L/min, auxiliary gas flow rate of 0.776 L/min, peristaltic pump rate of 40 r/min, retention time of 0.1 s. Compound identification was performed by retention time matching with quantification achieved through external calibration curves constructed from peak area integration.

### 2.3 Animal experiment design

**Figure 1:**
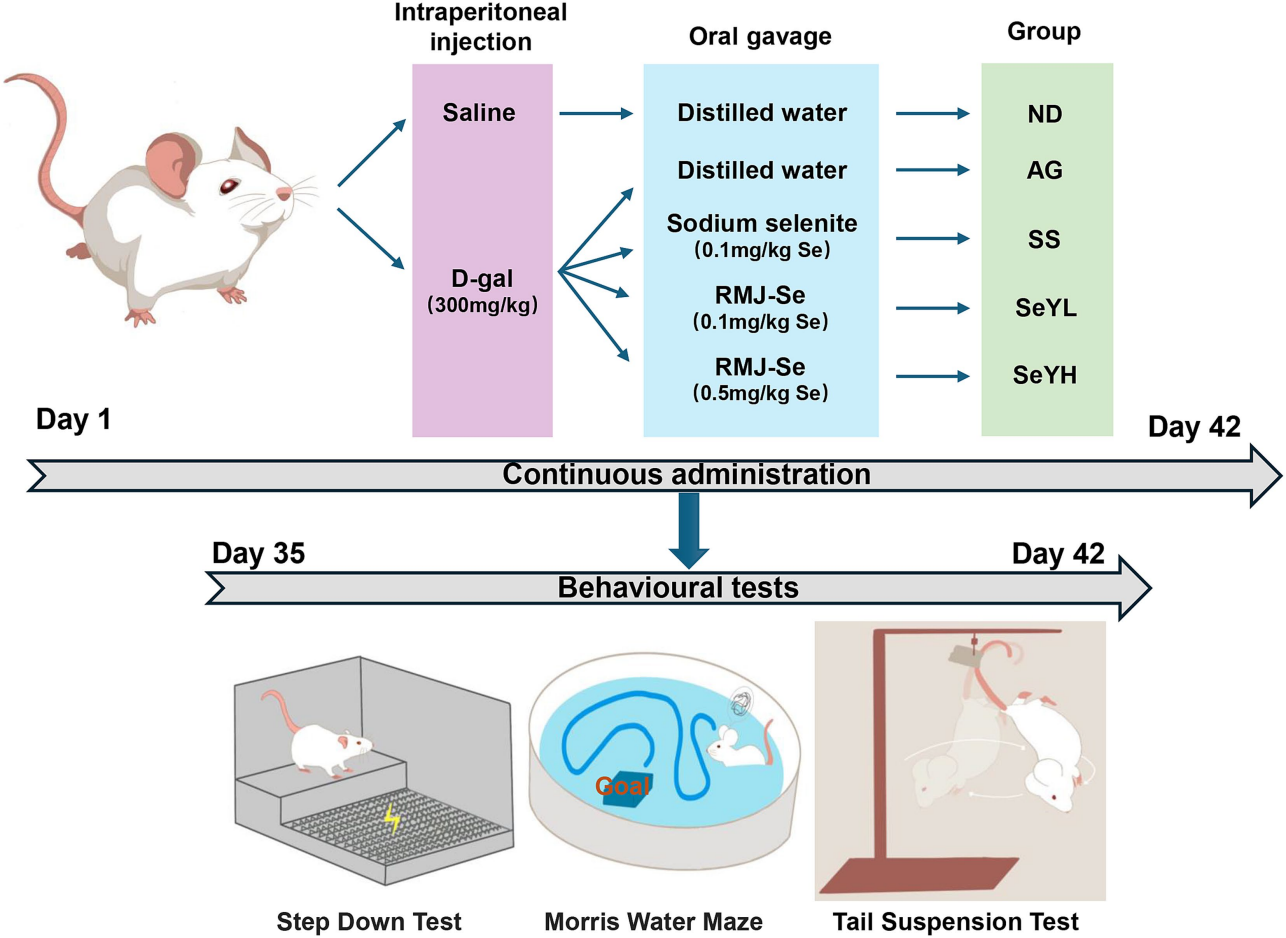
Animal experimental design.

Forty SPF-grade male Kunming mice (weight 18–22 g) were purchased from Liaoning Changsheng Biotech Co., China, and animal experiment was approved by the Animal Committee of Jilin Academy of Agricultural Sciences (LAMECJAAS-2022-016). To eliminate the potential influence of the female estrous cycle on oxidative stress indicators, inflammatory responses and gut microbial composition, only male mice were used in this study. The mice were reared at 25 °C with 12 h of light and 12 h of dark cycling. After 1 week of acclimatization, the mice were divided into ND, AG, SS, SeYL and SeYH groups, with 8 mice in each group. Mice in the ND group were gavaged with distilled water and intraperitoneally injected with saline; mice in the AG group were intraperitoneally injected with D-galactose and gavaged with distilled water; mice in the SS group, the SeYL group and the SeYH group were intraperitoneally injected with D-galactose, and gavaged with sodium selenite (selenium content of 0.1 mg/kg), a low-dose *Se-RMSRY* (selenium content of 0.1 mg/kg), and a high-dose *Se-RMSRY* (selenium content of 0.5 mg/ kg), respectively. The specific group and dose are shown in Figure 1. Intraperitoneal injections at a dose of 300 mg/kg bw and gavage at a dose of 0.1 mL/10 g bw were administered at the same time each day for 42 d. Body weights of the mice were recorded every 2 days and behavioral tests were performed at the end of 5 weeks.

### 2.4 Animal behavioral tests

#### 2.4.1 Tail suspension test

The device consisted of a wooden box 54 cm long and 30 cm wide. The hook was placed in the middle of the top of the wooden box, maintaining 350 mm from the lowermost part of the device. The experimental apparatus comprised hooks to which the tails of the mice were fastened, and the mice were observed for the final 4 minutes of their suspension. Small movements of only the forelimbs, but not the hindlimbs, or oscillations due to inertia, could be judged as stationary.

#### 2.4.2 Spatial acquisition trials

The experiment was conducted in a cylindrical water maze measuring 45 cm in diameter and 37 cm in depth, with an 8 cm-wide escape platform and a temperature of 23 °C ± 2 °C. At the beginning of each trial, the mouse was gently placed in the water at the designated location facing the edge of the circular pool. Mice were given 120 s to ascend to the platform and were allowed to remain on the platform for 10 s. In instances where a mouse failed to reach the platform within the allocated 120 s, it was gently guided towards the platform and allowed to remain there for a duration of 10 to 15 s. The mouse was not allowed to ascend to the platform. The ability of mice to memorize the platform position was assessed in terms of the time it took them to finally mount the platform (escape latency).

#### 2.4.3 Passive avoidance test

The mice were acclimatized to the reactor environment for a period of 3 min prior to the induction of the shock. The mice were then exposed to a charged enclosure (36 V alternating voltage) positioned at the base of the reactor. The experimental apparatus consisted of an electric shock chamber fitted with an insulated platform. During electric shock stimulation, the time taken for each mouse to escape onto the platform was recorded as the escape latency. After mounting the platform, mice might step down again. The time that mice remained on the platform before stepping down was defined as platform retention time, namely the interval from platform entry to the first subsequent step-down. If a mouse did not step down within 5 min, the latency was recorded as the maximum value of 300 s. Both escape latency and platform retention time were assessed in the training phase (day 1), retest phase (day 2), and memory retention phase (day 7).

### 2.5 Sample collection

At the end of the behavioral test, mice were anesthetized with isoflurane and blood was collected from the heart. Blood samples were centrifuged at 4 °C and 3,000 rpm for 10 min to obtain serum. Liver samples were homogenized to determine the biochemical parameters, including MDA, SOD, GSH-Px, AST, ALT (Nanjing Jiancheng Bioengineering Institute, China), TrxR, IL-6 and TNF-α (Beijing Rui Da Heng Hui Science and Technology Development Co. Ltd., China). MDA, SOD, GSH-Px, TrxR, IL-6, and TNF-α were determined after homogenization of jejunal samples. Liver, jejunum and brain tissues were fixed with paraformaldehyde for histopathological analysis. Some liver and jejunum tissues were frozen in liquid nitrogen to avoid light and stored at −80 °C for ROS fluorescence detection. The intestinal contents were frozen in liquid nitrogen and stored at −80 °C for 16sRNA sequencing.

### 2.6 Histopathological analysis

Mice tissues were embedded in paraffin, sectioned, stained with hematoxylin and eosin solution and examined under a microscope. Brain tissue was stained with Nysted and photographed with an Eclipse Ci-L (Nikon, Japan). The number of neurons in the images and the tissue area were recorded using Image-Pro Plus 6.0 software, and the number of neurons per unit area was calculated.

### 2.7 ROS fluorescence detection

Tissue sections were sent to Wuhan Servicebio Technology Co., Ltd. for ROS detection using fluorescence microscopy. Briefly, sections were prepared for ROS detection by several steps, including fluorescence burst, rinsing, CY3 incubation, decolorization in PBS, DAPI incubation, decolorization in PBS, and sealing with antifluorescence burst. DAPI excitation wavelength was 330–380 nm, emission wavelength was 420 nm. CY3 excitation wavelength was 510–560 nm, and emission wavelength was 590 nm.

### 2.8 Quantitative PCR analysis

Total liver RNA was extracted using a commercial kit (Cowin Biotech Co., Ltd., Jiangsu, China). qRT-PCR was performed using the QuantStudioTM 6 Flex Real-Time Fluorescence Quantitation System and a HiScript II One Step qRT-PCR Green Kit (Vazyme Biotechnology Co., Ltd., Jiangsu, China). The results were normalized to the expression of glyceralde hyde-3-phosphate dehydrogenase (GAPDH). The primer sequences were adopted from the research of Zhao et al. (2019).

### 2.9 Western blot

The liver and jejunum protein concentration were measured using a BCA kit (Nanjing Jiancheng Bioengineering Institute, China). The target protein was separated using SDS-PAGE (Epizyme Biomedical Technology Co., Ltd., China) and transferred to a polyvinylidene fluoride membrane (Millipore Inc., MA, USA). The tissue was sealed with 3% BSA for 1 h and then incubated with primary antibodies to Nrf2 (1:2,000), HO-1 (1:2,000), NQO1 (1:2,000), GCLC (1:2,000), GCLM (1:2,000), Bcl-2 (1:1,000), Bax (1:2,000) overnight at 4 °C. Goat anti-rabbit secondary antibodies were added, and the cells were incubated for 1 h. The samples were detected by chemiluminescence imaging. *β*-Actin (1:20,000) was used as a control. Primary antibodies were purchased from Proteintech Group, Inc., China.

### 2.10 16sRNA sequencing and gut microbiome analysis

Cecal contents were sent to Shanghai Personal Biotechnology Co., Ltd. for 16S rRNA detection. The raw sequencing data was processed on the Illumina NovaSeq platform. The 16S rRNA sequences have been uploaded to the China National Center for Bioinformatics (Project number: PRJCA058086). High-quality reads were classified into operational taxonomic units (OTUs) with 97% similarity using the DADA2 method for primer removal, quality filtering, denoise, splicing and chimera removal. Sequences were annotated and compositional distributions at the categorical level of each sample were counted using QIIME2 software, and Chao1, Shannon and Simpson indices were also calculated. Beta diversity was assessed using Principal Coordinates Analysis (PCoA). LDA Effect Size (LEfSe) was used to analyze representative microorganisms. Marker species were found using random forest modelling. PICRUSt2 software was used to predict the functional abundance of samples based on the Kyoto Encyclopedia of Genes and Genomes (KEGG) database to find differential pathways.

### 2.11 Statistical analysis

The results are presented as the mean ± standard error of the mean (sem). Statistics analysis was performed by using SPSS 24.0 and the figures were drawn by Origin 8.0 software. Statistical analysis was performed using one-way analysis of variance, followed by the least significant difference test. Differences were considered statistically significant at *p* < 0.05.

## 3 Results

### 3.1 Characterization of *se-RMSRY*

**Figure 2:**
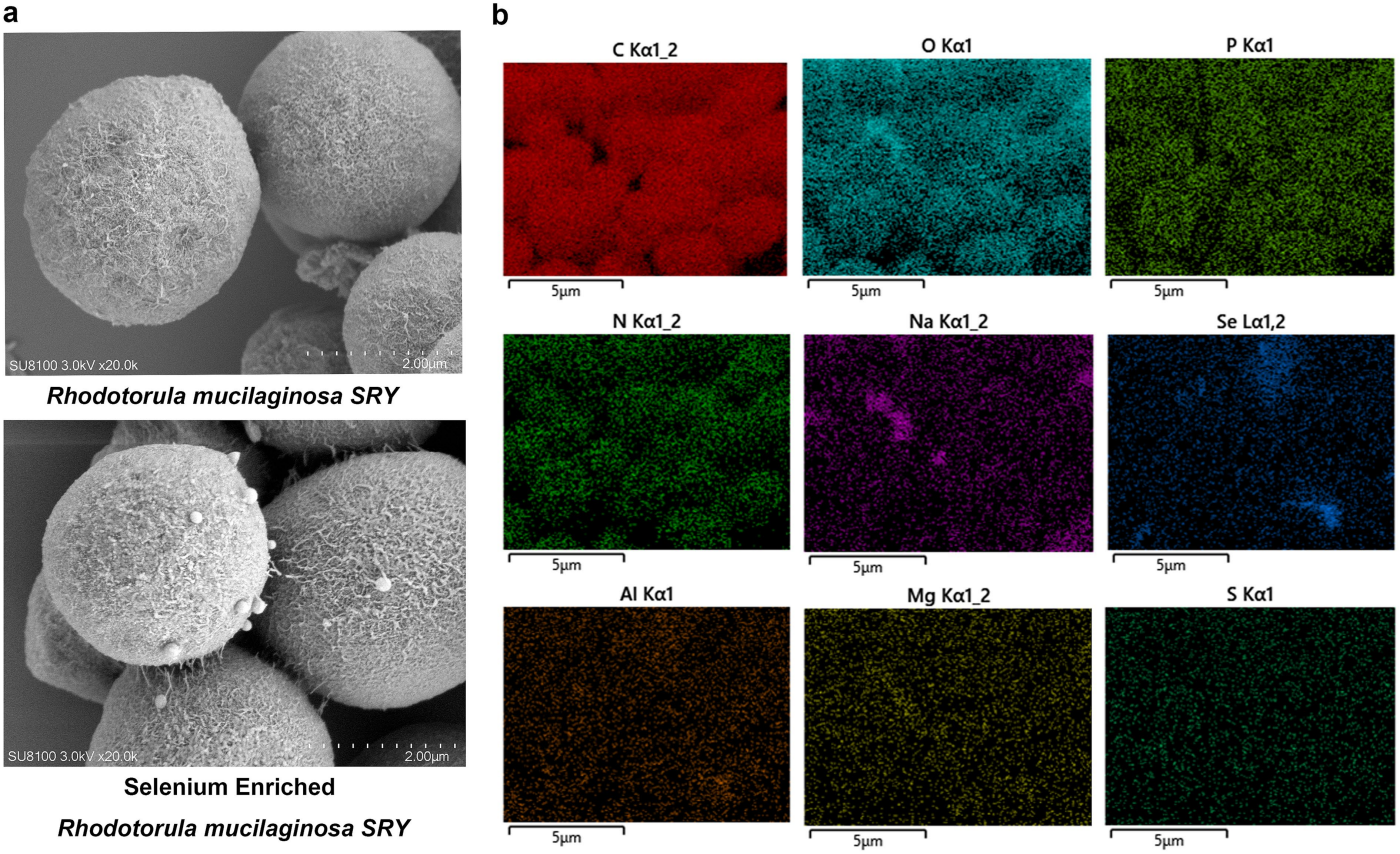
Characterization of *Se-RMSRY*. **(a)** Scanning electron microscopy, **(b)** elemental distribution of *Se-RMSRY.*

Scanning electron microscopy revealed that *Se*-*RMSRY* formed spherical microaggregates (Figure 2a), while energy-dispersive X-ray spectroscopy (EDS) elemental mapping of *Se-RMSRY* demonstrated preferential selenium enrichment within these structures, constituting 8.1% of the elemental composition, exceeding the concentrations of sodium (0.5%) and phosphorus (0.4%) concentrations (Figure 2b; Supplementary Figure 1). X-ray photoelectron spectroscopy (XPS) analysis confirmed SeMet as the predominant selenium species, with quantitative HPLC-MS measurements revealing a peak concentration of 1213.16 mg/kg (Table 1).

**Table 1:** Form and content of selenium.

Form	Content (mg/kg)
SeCys_2_	337.62 ± 20.74
SeMeCys	190.59 ± 21.68
SeMet	1213.16 ± 54.13
Se^4+^	256.33 ± 17.25
Unknown selenium form	135.02 ± 18.36
Insoluble selenium form	1048.35 ± 41.49

### 3.2 Effect of *se-RMSRY* on the growth status of aging mice

The mice in each group exhibited a gradual increase in body weight during the test. However, the endpoint body weight and the rate of body weight gain in the AG group were marginally lower than those in the other groups. In contrast, the SeYH group demonstrated the highest endpoint body weight and rate of body weight gain, along with the highest average water consumption and average food intake. Nonetheless, these differences among the groups did not reach statistical significance (*p* > 0.05, Supplementary Figure 2; Supplementary Table 1).

### 3.3 *Se-RMSRY* alleviated anxiety and improved learning and memory in mice

**Figure 3:**
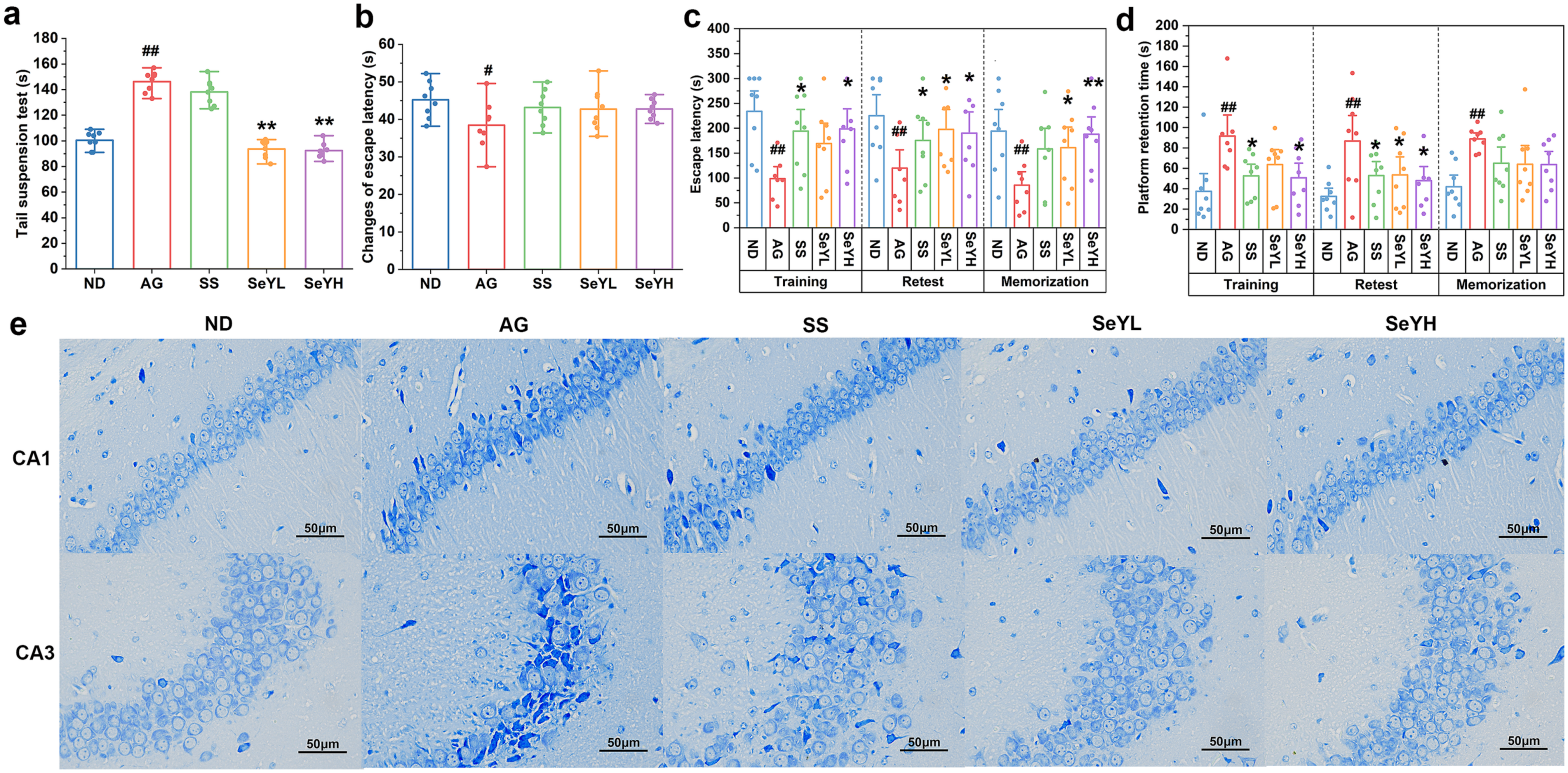
Effects of *Se-RMSRY* on cognition and oxidative state of the brain in mice. **(a)** Tail suspension test, **(b)** spatial acquisition trials, **(c)** escape latency of passive avoidance test, **(d)** platform retention time of passive avoidance test, **(e)** Nissl staining of neurons in hippocampal CA1 and CA3 area. The “#” represented a significant change compared to the ND group, *p* < 0.05. The “##” represented a highly significant change compared to the ND group, *p* < 0.01. The “*” represented a significant change compared to the AG group, *p* < 0.05. The “**” represented a highly significant change compared to the AG group, *p* < 0.01.

Prolonged over-oxidation of the brain has been demonstrated to induce a series of neurological abnormalities, including anxiety, depression and cognitive impairment. The effects of *Se-RMSRY* on the depressive state of aging mice were evaluated by the tail suspension test (Figure 3a). The results showed that the resting time of mice in the AG group was significantly longer than that in the ND group (*p* < 0.01). The resting time of mice in the SeYL and SeYH groups was significantly lower than that in the AG group (*p* < 0.01), and close to that of the ND group, implying that the depressive state of the aging mice had been alleviated.

The effects of *Se-RMSRY* on the learning ability and memory of mice were evaluated using the passive avoidance test and the spatial acquisition trials. During spatial acquisition trials, all experimental groups demonstrated progressive reductions in escape latency from day 2 onward, with the AG cohort exhibiting significantly prolonged escape latency durations relative to other groups (Supplementary Figure 3). A comparison of the changes in escape latency of mice in each group from day 1 to day 4 revealed that the changes in escape latency in the AG group were significantly lower than those in the ND group (*p* < 0.05). Furthermore, there were different degrees of increase in the SS, SeYL, and SeYH groups as compared with the AG group (*p* > 0.05, Figure 3b). These findings collectively suggest accelerated cognitive decline in AG mice, which was significantly mitigated in the SS, SeYL, and SeYH groups.

Consistent with cognitive deficits observed in spatial navigation tasks, AG mice displayed impaired performance in the passive avoidance test (Figures 3c,d). The AG group demonstrated significantly lower platform retention time and higher escape latency than the ND group across the three phases of training, retest, and memorization (*p* < 0.01). In contrast, platform retention time and escape latency of mice in the SS, SeYL and SeYH groups exhibited marked improvements, albeit to different extents.

### 3.4 *Se-RMSRY* alleviated oxidative damage status in mice brain

Hippocampal damage is associated with cognitive impairment in animals. In the ND group, the neuronal cells in the CA1 and CA3 regions of the hippocampus were arranged neatly and closely. In the AG group, the neuronal cells in the CA1 and CA3 regions of the hippocampus were arranged in a disordered way, and the neurons appeared to be atrophic, with obvious nuclear condensation, blurred boundaries of the nucleus membrane (Figure 3e). The number of neurons was significantly lower than that in the ND group (Supplementary Figure 4, *p* < 0.05). The SS, SeYL and SeYH groups, exhibited a substantial alleviation of hippocampal injury. Moreover, the SeYH group demonstrated a significant increase in the number of CA1 and CA3 neurons compared with the AG group (Supplementary Figure 4; *p* < 0.05).

### 3.5 *Se-RMSRY* improved hepatic oxidative stress and reduced inflammatory response

**Figure 4:**
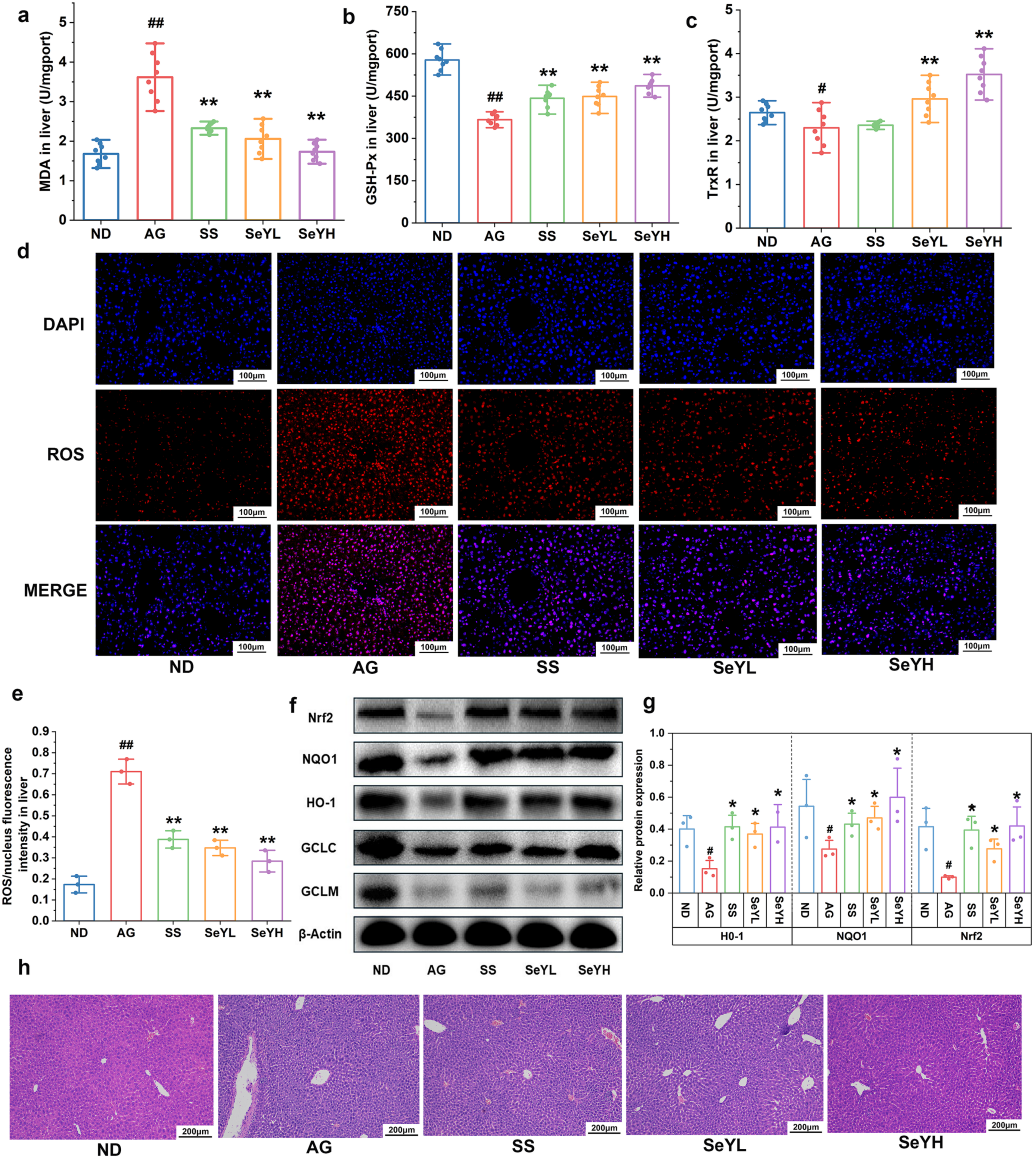
Effects of *Se-RMSRY* on the oxidative stress status of liver. **(a)** MDA, **(b)** GSH-Px, **(c)** TrxR, **(d)** ROS fluorescence staining, **(e)** ROS fluorescence intensity, **(f)** Western blot, **(g)** relative protein expression, **(h)** H&E staining of liver. The “#” represented a significant change compared to the ND group, *p* < 0.05. The “##” represented a highly significant change compared to the ND group, *p* < 0.01. The “*” represented a significant change compared to the AG group, *p* < 0.05. The “**” represented a highly significant change compared to the AG group, *p* < 0.01.

Hepatic inflammatory responses and redox imbalance are mechanistically linked to neurosystemic oxidative burden, resulting in significant behavioral alterations. Quantitative analysis revealed that AG mice showed 1.16-fold elevated hepatic MDA levels and 36.84% reduced GSH-Px activity compared with ND group (*p* < 0.01, Figures 4a,b). In contrast, the SS, SeYL and SeYH groups exhibited a marked decrease in hepatic MDA levels (*p* < 0.01, Figure 4a) and a significant increase in GSH-Px activity (*p* < 0.01, Figure 4b). The hepatic TrxR content in SeYL and SeYH groups was significantly higher than that in AG group (*p* < 0.01, Figure 4c). In addition, the hepatic ROS content in AG group was significantly higher than that in ND group, and was significantly reduced in SS, SeYL and SeYH groups compared with AG group (*p* < 0.01, Figures 4d,e). Western blot analysis of hepatic antioxidant pathways demonstrated marked downregulation of Nrf2 signaling components (Nrf2, NQO1, HO-1) and glutamate-cysteine ligase subunits (GCLC/GCLM) in AG group. These deficits were substantially rescued in SS, SeYL, SeYH groups (Figures 4f,g; Supplementary Figure 7). The above results indicated that the hepatic oxidative stress status was improved in mice in SS, SeYL and SeYH groups.

Sustained hepatic oxidative stress triggers a proinflammatory cascade, culminating in histopathological damage and functional impairment. The levels of hepatic inflammatory cytokines IL-6 and TNF-α were significantly increased in the AG group (*p* < 0.01). In contrast, IL-6 and TNF-α levels were significantly lower in the SeYL and SeYH group (*p* < 0.01, Supplementary Figure 5). There was no obvious tissue damage or pathological changes identified in the liver tissues. However, the AG group exhibited localized inflammatory cell infiltration in the liver, which was absent in SS, SeYL, and SeYH groups (Figure 4h). A comparison of liver function in mice revealed that the levels of ALT and AST in the SS and SeYL groups were significantly lower than those in the AG group (*p* < 0.01), with the most pronounced decrease observed in the SeYH group (Supplementary Figure 6).

### 3.6 *Se-RMSRY* improved intestinal oxidative stress and reduced inflammatory response

**Figure 5:**
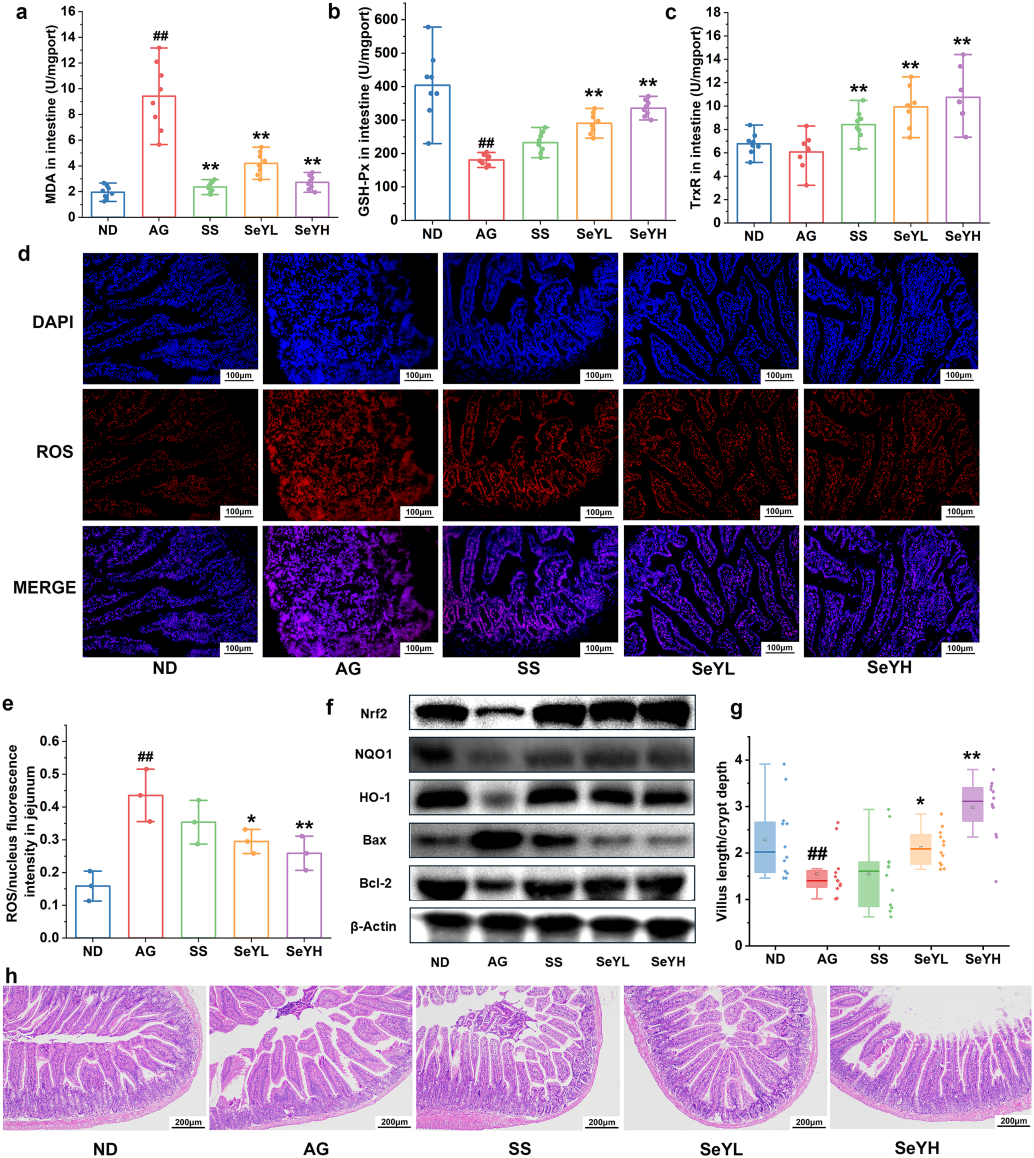
Effects of *Se-RMSRY* on the oxidative stress status of intestine. **(a)** MDA, **(b)** GSH-Px, **(c)** TrxR, **(d)** ROS fluorescence staining, **(e)** ROS fluorescence intensity, **(f)** Western blot, **(g)** Chorionic villus length to crypt depth ratio, **(h)** H&E staining of intestine. The “#” represented a significant change compared to the ND group, *p* < 0.05. The “##” represented a highly significant change compared to the ND group, *p* < 0.01. The “*” represented a significant change compared to the AG group, *p* < 0.05. The “**” represented a highly significant change compared to the AG group, *p* < 0.01.

Neurosystemic oxidative stress and associated behavioral deficits (including anxiety-like behaviors and memory impairment) were mechanistically linked to intestinal redox imbalance and inflammatory cascades via the gut-brain axis. As shown in Figures 5a,b, MDA levels in the small intestine of mice in the AG group were highly significantly increased, and GSH-Px activity was significantly decreased (*p* < 0.01). Compared with the AG group, the SS, SeYL and SeYH groups showed significant decreases in MDA levels, and the SeYL and SeYH groups exhibited significant increases in GSH-Px activity and TrxR content (*p* < 0.01; Figures 5a−c). Compared with the ND group, the jejunal ROS intensity was significantly elevated in the AG group of mice (*p* < 0.01). Conversely, the ROS intensity was significantly reduced in the SeYL and SeYH groups relative to the AG group (*p* < 0.05, Figures 5d,e). Furthermore, compared with the AG group, the mRNA expression levels of Nrf2, NQO1, and HO-1 were significantly increased in the SS, SeYL, and SeYH groups (Supplementary Figure 8). The AG group exhibited a significant decrease in oxidative stress-related proteins Nrf2 and HO-1 (*p* < 0.05), with NQO1 displaying a downward trend (*p* > 0.05). However, this decline was mitigated to varying extents in the SS, SeYL and SeYH groups (Figure 5f; Supplementary Figure 8). The results indicated that *Se-RMSRY* could alleviate the oxidative stress state in the jejunum of mice.

In the subsequent phase of the study, the effects of *Se-RMSRY* on intestinal inflammation and injury were investigated. IL-6 and TNF-α levels in the intestine were significantly higher in the AG group than in the ND group (*p* < 0.01). Compared with the AG group, IL-6 levels were significantly lower in the SeYL and SeYH groups (*p* < 0.01), and TNF-α levels were significantly lower in the SS, SeYL and SeYH groups (*p* < 0.01, Supplementary Figure 5).

H&E staining of the jejunum showed that the villi in the ND group were arranged orderly and densely packed, in contrast to the sparse and disorganized state of the jejunal villi in the AG group. The AG group also exhibited a significantly larger lumen area, with some villi displaying severe detachment and apical breakage (Figure 5h). Compared with the AG group, the intestinal villi in the SS, SeYL and SeYH groups were more complete in morphology, and the ratio of villus length to crypt depth was significantly increased (*p* < 0.05, Figure 5g). In addition, the expression of pro-apoptotic protein Bax tended to be elevated in the AG group (*p* > 0.05), compared with a significant decrease in the protein expression of Bax in the SeYL and SeYH groups (*p* < 0.05, Figure 5f; Supplementary Figure 8). The protein expression of Bcl-2 in the AG group was significantly lower than that in the ND group (*p* < 0.01), and that in the SS, SeYL, SeYH groups was significantly higher than that in the AG group (*p* < 0.05, Figure 5f; Supplementary Figure 8). The above results indicated that *Se-RMSRY* alleviated the intestinal villi damage and inflammatory response caused by oxidative stress in mice.

### 3.7 *Se-RMSRY* regulated gut microbiota balance in mice

**Figure 6:**
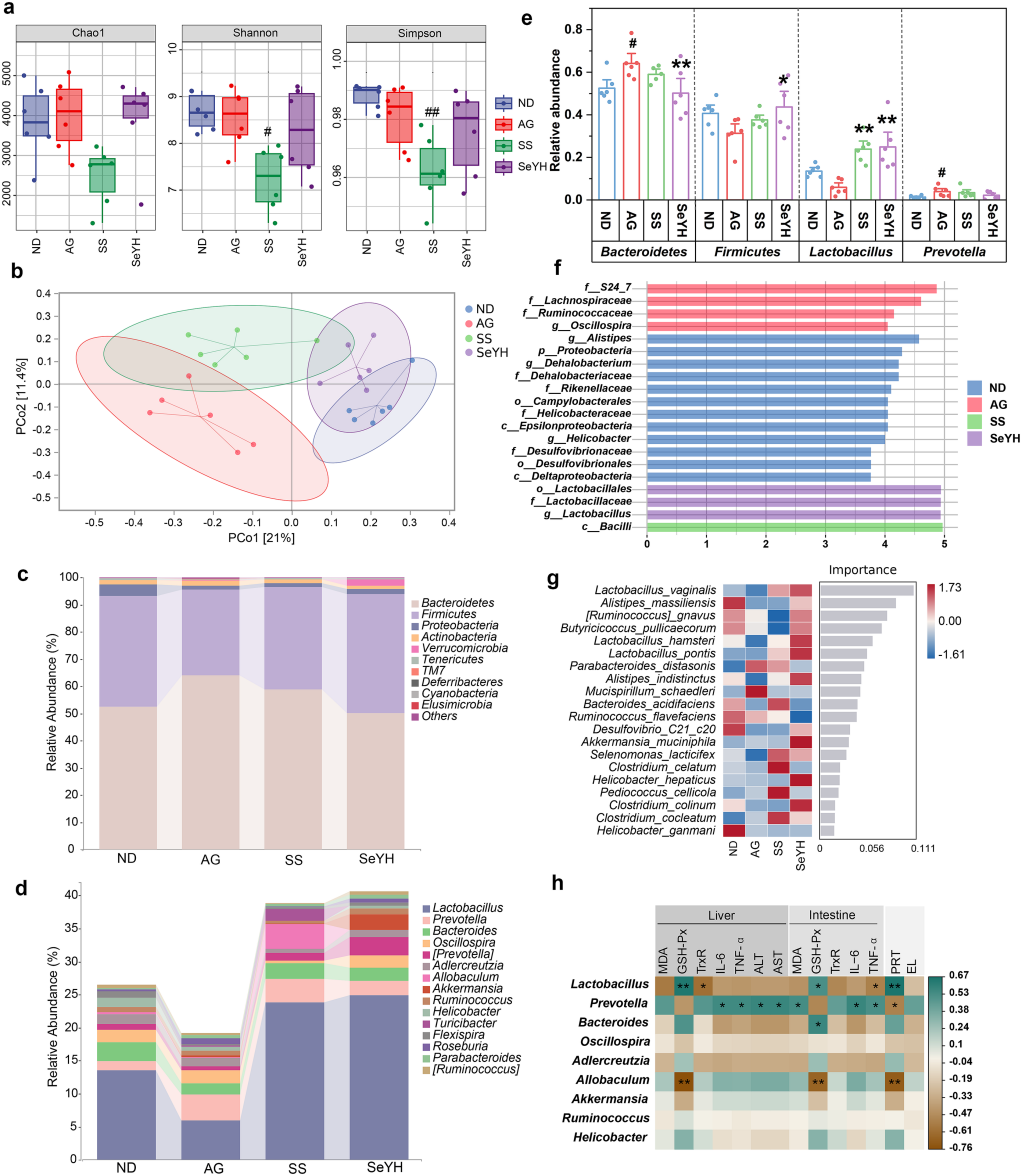
Effect of *Se-RMSRY* on the gut microbiota. **(a)** *α*-Diversity, **(b)** β-diversity, **(c)** Gut microbial differences at the phylum level, **(d)** Gut microbial differences at the genus level, **(e)** Differential microbial relative abundance, **(f)** LEfSe, **(g)** Random forest prediction of marker microorganisms in the gut, **(h)** Correlation analyses. The “#” represented a significant change compared to the ND group, *p* < 0.05. The “##” represented a highly significant change compared to the ND group, *p* < 0.01. The “*” represented a significant change compared to the AG group, *p* < 0.05. The “**” represented a highly significant change compared to the AG group, *p* < 0.01.

Since high-dose *Se-RMSRY* was more effective than low-dose *Se-RMSRY* in ameliorating oxidative damage in mice, a subsequent investigation was undertaken to analyze the impact of high-dose *Se-RMSRY* on the gut microbiota. As shown in Figure 6a, the variation in α-diversity among the ND, AG and SeYH groups was not statistically significant. However, a significant decrease was observed in the SS group (*p* < 0.05), indicating that sodium selenite diminished the abundance of intestinal microbiota in mice. PCoA was utilized to demonstrate the composition and distribution of the mice gut microbiota, in which the SeYH group was completely separated from the AG group and closer to the gut microbial composition of the ND group (Figure 6b). Further analysis of the differences in the community composition of the mice gut microbiota among the groups revealed that at the phylum level, the relative abundance of *Bacteroidetes* was significantly increased (*p* < 0.05) and the relative abundance of *Firmicutes* was decreased in the AG group compared with the ND group, and this trend was reversed in SeYH (Figures 6c,e). At the genus level, the relative abundance of *Lactobacillus* sp. was markedly elevated in both the SS and SeYH groups compared with the AG group (*p* < 0.01, Figures 6d,e). LEfSe analysis was further applied to screen microbial biomarkers with significant differences among the groups. *Alistipes* sp.*, Dehalobacterium* sp. and *Helicobacter* sp. served as the characteristic taxon of the ND group, while *Oscillospira* sp. was identified as the characteristic taxon distinguishing the AG group. Of note, *Lactobacillus* sp. was pinpointed as a unique biomarker specific to the SeYH group (Figure 6f). Random forest modeling was further used to screen intestinal marker species in mice. The results showed that *Lactobacillus vaginalis, Lactobacillus hamsteri,* and *Lactobacillus pontis* exhibited high importance and contribution in the SeYH group (Figure 6g).

**Figure 7:**
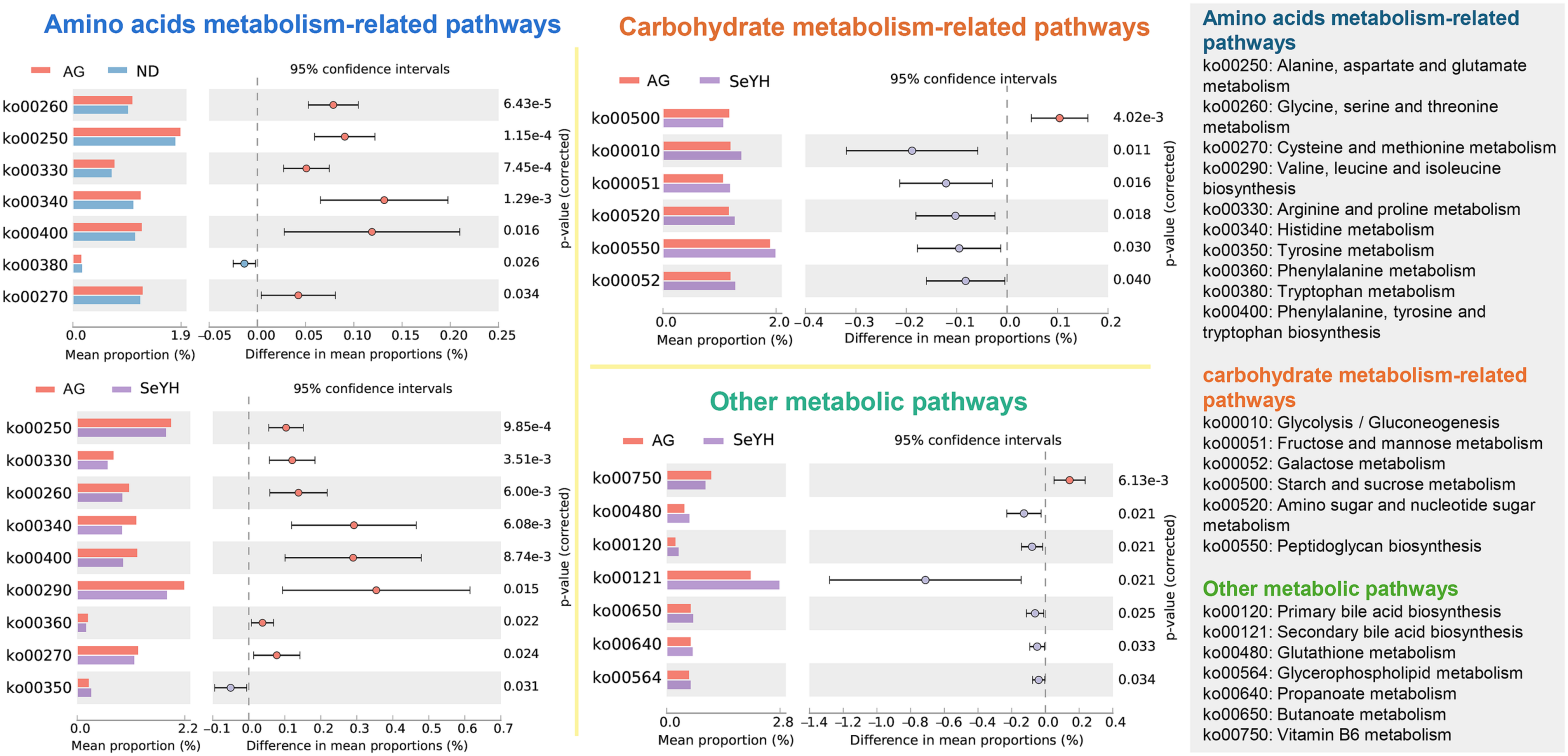
Differential metabolic pathway enrichment analysis.

An analysis of the differences in metabolic pathways among the groups based on gut microbiota revealed that pathways related to amino acid metabolism were significantly upregulated in the AG group compared to the ND group (*p* < 0.05, Figure 7). The SeYH group was not only significantly downregulated in amino acid metabolism but also more active in carbohydrates metabolism compared to the AG group (*p* < 0.05). Furthermore, the SeYH group exhibited a significant upregulation in pathways related to glutathione metabolism, bile acid biosynthesis, and short-chain fatty acid metabolism (*p* < 0.05).

### 3.8 Correlation analysis

Further analysis of the correlations among the gut microbiota, oxidative levels, inflammatory cytokines in the liver and jejunum, as well as cognitive behavior in mice, showed that *Lactobacillus* sp. was significantly negatively correlated with MDA and TrxR levels in the liver and TNF-α levels in the gut (*p* < 0.05), and significantly positively correlated with GSH-Px activity in the liver and jejunum (*p* < 0.05), as well as with platform retention time in the behavioral test. Furthermore, *Prevotella* sp. displayed a significant positive correlation with IL-6, TNF-α, ALT and AST levels in the liver, GSH-Px activity and TNF-α levels in the jejunum, and a significant negative correlation with step through latency (*p* < 0.05). *Allobaculum* sp. exhibited a highly significant negative correlation with GSH-Px activity in the liver and jejunum (*p* < 0.01, Figure 6h).

## 4 Discussion

The aging process is frequently accompanied by oxidative stress, organ degeneration and gut microbiota dysbiosis (Wang et al., 2021). A plethora of substances have been identified as regulators of the body’s redox system, including pigments, minerals, vitamins, and probiotics (Bjørklund et al., 2022). Selenium is a prevalent dietary supplement, renowned for its antioxidant properties. Selenium-enriched yeast accumulates exogenous selenium via uptake mechanisms and transports it inside the cell, where it undergoes a series of processes, including enzymatic degradation, oxidation, reduction, and methylation, ultimately leading to the formation of SeMet, and other selenoproteins (Kayrouz et al., 2022). In this study, the adsorption of selenium by *Rhodotorula mucilaginosa* JAASRY1 was observed on the cell surface, with subsequent conversion to SeMet, SeMeCys, and SeCys2. These selenoamino acids have been shown to promote the synergistic function of various selenoproteins in animals and to participate in the regulation of metabolic activities (Jing et al., 2023).

Oxidative stress is a significant factor in the acceleration of cellular senescence, a process characterized by ROS accumulation, which in turn causes substantial damage to DNA, proteins, and lipids (Maldonado et al., 2023). In this study, we found that both sodium selenite and *Se-RMSRY* reduced ROS levels in aging mice and significantly improved in MDA levels, GSH-Px activity, and TrxR content in liver and jejunum, suggesting that both can improve antioxidant capacity in aging mice. Sun et al. revealed that selenium-enriched yeast could enhance antioxidant capacity by increasing serum GSH-Px activity in dairy cows (Sun et al., 2021). As the primary antioxidant defense mechanism against ROS-induced oxidative stress, GSH-Px functions by reducing cellular oxidation levels through the conversion of hydrogen peroxide (formed from oxygen radical anions) back into water (Jomova et al., 2024). TrxR1 has been demonstrated to reduce thioredoxin, as well as other substrates, thus contributing to redox homeostasis by reducing ROS levels (Rong et al., 2021). As the immune system ages, it becomes incapable of eliminating senescent cells and pro-inflammatory factors. This, in turn, results in the gradual accumulation of low-grade chronic inflammation, which further exacerbates the aging process, thereby establishing a vicious cycle. In the present study, a significant increase in IL-6 and TNF-α levels was observed in the liver and jejunum of D-galactose-induced aging mice (*p* < 0.05). IL-6 and TNF-α are two senescence-associated secreted phenotypic factors (SASPs). The release of SASPs marks the initiation of systemic chronic inflammation, which propels cells into a senescent state (Li et al., 2023). *Se-RMSRY* (particularly at high doses) significantly suppressed both pro-inflammatory cytokines, demonstrating potent attenuation of senescence-associated chronic inflammation. Furthermore, the study demonstrated that chronic oxidative stress and inflammation can lead to senescent injury in multiple organs throughout the body, a condition that was particularly evident in the jejunum in this study. Sodium selenite and *Se-RMSRY* were observed to slow down intestinal villus injury in mice. In the liver, aging mice exhibited elevated levels in enzyme systems associated with liver function, which were reduced to varying extents by sodium selenite and *Se-RMSRY*. *Rhodotorula mucilaginosa* has been proven to reduce the level of oxidative stress in the body and enhance anti-inflammatory capacity, thereby alleviating pathological damage and inflammatory infiltration in tissues.

A further characteristic manifestation of the aging process, attributable to over-oxidation, is the deterioration of memory and cognitive abilities. This study demonstrated that both *Se-RMSRY* and sodium selenite ameliorated these effects by enhancing neuronal proliferation in the hippocampus of mice. The number of neurons in the hippocampus has been observed to decrease with age, resulting in impaired cognitive function (Denoth-Lippuner and Jessberger, 2021). Drapeau et al. (2003) indicated that an increase in neurogenesis was associated with improved behavior dependent on the hippocampus, as measured by a spatial acquisition trial. Low dietary doses of selenium showed antioxidant activity in the brain. A study by Zhang et al. found that selenium yeast significantly improved spatial learning and memory, promoted neuronal activity, and outperformed SeMet due to the multitude of elements, vitamins and other nutrients present in selenium yeast (Zhang et al., 2017; Zhang et al., 2018).

In this study, the antioxidant effect of *Se-RMSRY* was generally superior to that of sodium selenite at the same selenium concentration. This phenomenon may be attributed to two potential factors. Firstly, *Se-RMSRY* produces selenoamino acids that are more efficiently utilized by the body, whereas the selenium present in sodium selenite requires biotransformation, a process that demands consumes energy and amino acid substrates (Nie et al., 2023). Secondly, *Rhodotorula mucilaginosa* biosynthesizes potent antioxidant pigments, including *β*-carotene and astaxanthin, which have demonstrated free radical scavenging capacities. The yeast’s cell wall components, mannoproteins and β-glucans can modulate immune responses, whereas selenium incorporation has also contributed to enhanced catalytic and oxidoreductase activity of *Rhodotorula mucilaginosa* (Díaz-Navarrete et al., 2024).

Building on the potent antioxidant efficacy of *Se-RMSRY*, the study investigated its molecular targets within the Nrf2/ARE pathway. Nrf2 is a major sensor of oxidative stress that regulates redox homeostasis. When cells are stimulated by stress, a conformational change in Keap1 leads to its uncoupling from Nrf2, which in turn induces Nrf2 to bind to the ARE and regulate downstream transcription of antioxidant genes such as HO-1 and NQO1 (De Gaetano et al., 2021). HO-1 catalyzes the catabolism of heme to biliverdin, which is effective in scavenging ROS. NQO1 reduces the production and toxic effects of ROS by catalyzing the reduction of quinones and the covalent binding of glutathione. Moreover, Nrf2 inhibits the activation of the NF-κB signaling pathway, thereby attenuating inflammatory damage in tissue cells (Casper, 2023). Nrf2 can upregulate the expression of the Bcl-2 family of anti-apoptotic proteins, further inhibiting the apoptotic process (Tian et al., 2012). In the present study, *Se-RMSRY* was observed to induce a substantial augmentation in jejunal Bcl-2 protein expression levels in aging mice. The Bcl-2 protein has been shown to form a heterodimer with Bax, thereby blocking the release of Cyt-c and inhibiting the activation of Caspase-3 (Czabotar and Garcia-Saez, 2023). Concurrently, Bcl-2 has been observed to inhibit Apaf-1 and prevent the activation of Caspase-9, thus effectively inhibiting apoptosis (Czabotar and Garcia-Saez, 2023). Ju et al. (2021) demonstrated that SeMet potently activated Nrf2 nuclear translocation and upregulated downstream targets, exhibiting superior efficacy to sodium selenite in both Nrf2 pathway activation and reduction of the apoptotic index. Beyond the Nrf2/ARE pathway, emerging evidence has identified the thioredoxin-interacting protein (TXNIP)/NLRP3 inflammasome axis as a critical molecular link bridging oxidative stress to inflammatory responses (Abais et al., 2014; Datta et al., 2025). In this paradigm, excessive ROS production triggers TXNIP to dissociate from thioredoxin (Trx) and bind to NLRP3, leading to inflammasome assembly, caspase-1 activation, and subsequent maturation of IL-1β and IL-18. In our study, we observed that D-galactose-induced aging significantly decreased TrxR activity, which is consistent with the activation of this pathway. These findings align closely with our data showing SeMet-enhanced antioxidant enzyme activities.

The gastrointestinal tract serves as the primary site for selenium absorption and metabolism, with selenium supplementation significantly modulating murine gut microbiota diversity. Zhai et al. (2018) demonstrated that dietary selenium supplementation modulated gut microbiota composition and enhanced colonic selenoprotein expression in mice. *Rhodotorula mucilaginosa* enhances gut microbial homeostasis by promoting beneficial taxa such as *Lactobacillus* sp. while suppressing pathogenic populations. Studies have shown that *Rhodotorula mucilaginosa* improves immune function and gut microbiota by increasing the presence of beneficial bacteria such as *Lactobacillus* sp., while decreasing the presence of harmful bacteria such as *Bacteroidetes* (Chen et al., 2019; Ge et al., 2021). The present study corroborated these findings. In this study, *Se-RMSRY* and sodium selenite significantly increased the relative abundance of *Lactobacillus* sp. in the intestinal tracts of mice (*p* < 0.01). *Lactobacillus vaginalis* emerged as the predominant modulator of gut microbial dynamics, with *Lactobacillus hamsteri* and *Lactobacillus pontis* exerting complementary functional roles. As posited by Wu et al., an analysis of the gut microbiota revealed that the subjects in the longevity group exhibited elevated levels of microbial diversity, exogenous biodegradation and metabolism, and oxidoreductases. Furthermore, the gut antioxidant system was found to be associated with the high antioxidant activity of the *Lactobacillus* sp. (Wu et al., 2022)*. Lactobacillus* sp. produces gamma-aminobutyric acid (GABA), which acts as an inhibitory neurotransmitter connecting the gut to the central nervous system, and regulates mental health and cognitive performance (Braga et al., 2024). Furthermore, KEGG pathway analysis of the gut microbiota revealed that amino acid metabolism was more active in aging mice, whereas carbohydrate metabolism was more active in the Se-RMSRY group. Recent studies have indicated that the majority of amino acids exhibit an increase with age (except tryptophan), a phenomenon that may be associated with an elevated catabolic rate of these compounds (Darst et al., 2019; Lawton et al., 2008; Panyard et al., 2022). The present study obtained similar results, with *Se-RMSRY* exhibiting resistance to aging while concomitantly reducing tryptophan metabolism. Gut microbes are involved in cognitive and memory behaviors by regulating tryptophan metabolism, and their metabolites also modulate inflammatory cytokines to maintain gut homeostasis (Xue et al., 2023; Zhang et al., 2022). Through microbial hydrolysis, dietary polysaccharides are catabolized to mono−/oligosaccharides, which are channeled into distinct bacterial metabolic pathways. Subsequent anaerobic fermentation yields short-chain fatty acids that orchestrate multifaceted physiological responses including oxidation, inflammation, energy metabolism, and intestinal barrier (Mann et al., 2024). It was hypothesized that *Se-RMSRY* might play a role in combating aging by modulating microbial carbohydrate metabolism in the gut. This, in turn, might regulate the production of short-chain fatty acids. Moreover, *Se-RMSRY* was found to upregulated the glutathione metabolic pathway in the intestines of aging mice, suggesting that the oxidative defense mechanisms were enhanced (Benjamin et al., 2023; Panyard et al., 2022).

Following intestinal absorption, dietary components undergo microbial biotransformation, generating bioactive metabolites that exert systemic regulatory effects on extraintestinal organs. The leakage of harmful bacterial metabolites from the gut leads to bacterial and endotoxin translocation, creating systemic low-grade inflammation and oxidative stress, which promote substantial lesions in other organs (Tilg et al., 2022). In a similar manner, hepatic secretory functions can exert a feedback effect on the gut, thereby affecting the microbiota, epithelial cells, and immune cells, and consequently triggering further repair of gut function (Pabst et al., 2023). However, further research is required to explore the role of gut microbial factors in regulating or being regulated by liver function (Pabst et al., 2023). Simultaneously, microorganisms can also induce enteroendocrine cells to secrete 5-hydroxytryptamine and other cellular hormones, and bind to synaptic receptors, thereby eliciting electrical impulses that modulate synaptic transmission via nerve terminals and afferent vagal fibers (Akram et al., 2024; Honarpisheh et al., 2022). Consequently, the signal is transmitted to the central nervous system, thereby regulating mood, memory, and cognition, as well as maintaining hormonal balance and neurotransmitter release in enteroendocrine cells (Honarpisheh et al., 2022). Microbiota metabolites, including short-chain fatty acids, tryptophan derived indoles, and secondary bile acids, enhance blood–brain barrier and intestinal epithelial integrity by suppressing inflammatory cytokines (Agirman et al., 2021; Honarpisheh et al., 2022). This study revealed that *Se-RMSRY* and sodium selenite exerted therapeutic benefits by synergistically restoring intestinal barrier integrity and remodeling gut microbial homeostasis. Certain protective probiotics (e.g., *Lactobacillus* sp.) have been shown to stimulate the host immune system, thereby counteracting the deleterious effects of compromised intestinal barrier function. This intervention facilitated the restoration of hepatic and neurological functions through bidirectional crosstalk along the gut-liver and gut-brain axes, while coordinately enhancing systemic antioxidant defenses to mitigate age-related physiological decline.

## 5 Conclusion

This study demonstrated that *Se-RMSRY*, containing SeMet, SeCys2, and SeMeCys, exhibited superior anti-aging efficacy over inorganic sodium selenite by orchestrating multi-organ protective mechanisms. *Se-RMSRY* restored intestinal homeostasis through enrichment of beneficial *Lactobacillus* sp. (*L. vaginalis, L. hamsteri, L. pontis*), favoring carbohydrate utilization over amino acid metabolism. Systemic antioxidant capacity was enhanced via activation of the Nrf2/NQO1/HO-1 pathway and glutathione metabolism, reducing oxidative stress in intestinal and hepatic tissues. Furthermore, *Se-RMSRY* mitigated neuronal loss in the hippocampus and alleviated anxiety-like behaviors, likely mediated through gut-brain axis crosstalk, while concurrently suppressing apoptosis via Bcl2/Bax signaling modulation. These findings establish Se-RMSRY as a multifunctional dietary intervention, providing mechanistic insights for its application in functional foods targeting age-related oxidative stress, gut dysbiosis, and neurodegeneration.

## Data Availability

The datasets presented in this study can be found in online repositories. The names of the repository/repositories and accession number(s) can be found in the article/Supplementary material.

## References

[ref1] Abais J. M. Xia M. Li G. Chen Y. Conley S. M. Gehr T. W. B. . (2014). Nod-like receptor protein 3 (NLRP3) inflammasome activation and podocyte injury via thioredoxin-interacting protein (TXNIP) during hyperhomocysteinemia. J. Biol. Chem. 289, 27159–27168. doi: 10.1074/jbc.M114.567537, 25138219 PMC4175351

[ref2] Agirman G. Yu K. B. Hsiao E. Y. (2021). Signaling inflammation across the gut-brain axis. Science 374, 1087–1092. doi: 10.1126/science.abi6087, 34822299

[ref3] Akram N. Faisal Z. Irfan R. Shah Y. A. Batool S. A. Zahid T. . (2024). Exploring the serotonin-probiotics-gut health axis: a review of current evidence and potential mechanisms. Food Sci. Nutr. 12, 694–706. doi: 10.1002/fsn3.3826, 38370053 PMC10867509

[ref4] Benjamin D. I. Brett J. O. Both P. Benjamin J. S. Ishak H. L. Kang J. . (2023). Multiomics reveals glutathione metabolism as a driver of bimodality during stem cell aging. Cell Metab. 35, 472–486.e6. doi: 10.1016/j.cmet.2023.02.001, 36854304 PMC10015599

[ref5] Bjørklund G. Shanaida M. Lysiuk R. Butnariu M. Peana M. Sarac I. . (2022). Natural compounds and products from an anti-aging perspective. Molecules 27:7084. doi: 10.3390/molecules27207084, 36296673 PMC9610014

[ref6] Braga J. D. Thongngam M. Kumrungsee T. (2024). Gamma-aminobutyric acid as a potential postbiotic mediator in the gut-brain axis. npj Sci. Food 8:16. doi: 10.1038/s41538-024-00253-2, 38565567 PMC10987602

[ref7] Casper E. (2023). The crosstalk between Nrf2 and NF-κB pathways in coronary artery disease: can it be regulated by SIRT6? Life Sci 330:122007. doi: 10.1016/j.lfs.2023.122007, 37544377

[ref8] Che X. Shang X. WeiXu Xing M. Wei H. Li W. . (2025). Selenium-enriched *Lactiplantibacillus plantarum* alleviates alkalinity stress-induced selective hepatic insulin resistance in common carp. Int. J. Biol. Macromol. 305:141204. doi: 10.1016/j.ijbiomac.2025.141204, 39986514

[ref9] Chen F. Wang Y. Wang K. Chen J. Jin K. Peng K. . (2023). Effects of *Litsea cubeba* essential oil on growth performance, blood antioxidation, immune function, apparent digestibility of nutrients, and fecal microflora of pigs. Front. Pharmacol. 14:1166022. doi: 10.3389/fphar.2023.1166022, 37465523 PMC10350539

[ref10] Chen X. Q. Zhao W. Xie S. W. Xie J. J. Zhang Z. H. Tian L. X. . (2019). Effects of dietary hydrolyzed yeast (*Rhodotorula mucilaginosa*) on growth performance, immune response, antioxidant capacity and histomorphology of juvenile Nile tilapia (*Oreochromis niloticus*). Fish Shellfish Immunol. 90, 30–39. doi: 10.1016/j.fsi.2019.03.068, 31004799

[ref11] Cryan J. F. O'Riordan K. J. Sandhu K. Peterson V. Dinan T. G. (2020). The gut microbiome in neurological disorders. Lancet Neurol 19, 179–194. doi: 10.1016/s1474-4422(19)30356-4, 31753762

[ref12] Czabotar P. E. Garcia-Saez A. J. (2023). Mechanisms of BCL-2 family proteins in mitochondrial apoptosis. Nat. Rev. Mol. Cell Biol. 24, 732–748. doi: 10.1038/s41580-023-00629-4, 37438560

[ref13] D'Amato R. Regni L. Falcinelli B. Mattioli S. Benincasa P. Dal Bosco A. . (2020). Current knowledge on selenium biofortification to improve the nutraceutical profile of food: a comprehensive review. J. Agric. Food Chem. 68, 4075–4097. doi: 10.1021/acs.jafc.0c00172, 32181658 PMC7997367

[ref14] Darst B. F. Koscik R. L. Hogan K. J. Johnson S. C. Engelman C. D. (2019). Longitudinal plasma metabolomics of aging and sex. Aging (Albany NY) 11, 1262–1282. doi: 10.18632/aging.101837, 30799310 PMC6402508

[ref15] Datta S. Rahman M. A. Koka S. Boini K. M. (2025). Mitigation of nicotine-induced podocyte injury through inhibition of thioredoxin interacting protein. Biomed. Pharmacother. 187:118110. doi: 10.1016/j.biopha.2025.118110 40311224 PMC13569405

[ref16] De Gaetano A. Gibellini L. Zanini G. Nasi M. Cossarizza A. Pinti M. (2021). Mitophagy and oxidative stress: the role of aging. Antioxidants (Basel) 10:794. doi: 10.3390/antiox10050794, 34067882 PMC8156559

[ref17] Denoth-Lippuner A. Jessberger S. (2021). Formation and integration of new neurons in the adult hippocampus. Nat. Rev. Neurosci. 22, 223–236. doi: 10.1038/s41583-021-00433-z, 33633402

[ref18] Díaz-Navarrete P. Sáez-Arteaga A. Marileo L. Alors D. Correa-Galeote D. Dantagnan P. (2024). Enhancing selenium accumulation in *Rhodotorula mucilaginosa* strain 6S using a proteomic approach for Aquafeed development. Biomolecules 14:629. doi: 10.3390/biom14060629, 38927033 PMC11201420

[ref19] Drapeau E. Mayo W. Aurousseau C. Le Moal M. Piazza P. V. Abrous D. N. (2003). Spatial memory performances of aged rats in the water maze predict levels of hippocampal neurogenesis. Proc. Natl. Acad. Sci. USA 100, 14385–14390. doi: 10.1073/pnas.2334169100 14614143 PMC283601

[ref20] Ferrucci L. Gonzalez-Freire M. Fabbri E. Simonsick E. Tanaka T. Moore Z. . (2020). Measuring biological aging in humans: a quest. Aging Cell 19:e13080. doi: 10.1111/acel.13080, 31833194 PMC6996955

[ref21] Gao L. Li X. Li Y. Zhang Z. Wang J. Xu C. . (2024). Biochemical characterization, biosynthesis mechanism, and functional evaluation of selenium-enriched *aspergillus oryzae* A02. Int. J. Biol. Macromol. 275:133714. doi: 10.1016/j.ijbiomac.2024.133714, 38977051

[ref22] Ge Y. Huang K. Xie W. Xu C. Yao Q. Liu Y. (2021). Effects of *Rhodotorula mucilaginosa* on the immune function and gut microbiota of mice. Front. Fungal Biol. 2:705696. doi: 10.3389/ffunb.2021.705696, 37744147 PMC10512290

[ref23] Guo X. Li C. Zhang J. Sun M. Xu J. Xu C. . (2023). Chiral nanoparticle-remodeled gut microbiota alleviates neurodegeneration via the gut-brain axis. Nat. Aging 3, 1415–1429. doi: 10.1038/s43587-023-00516-9, 37946041

[ref24] Hamidi M. Gholipour A. R. Delattre C. Sesdighi F. Mirzaei Seveiri R. Pasdaran A. . (2020). Production, characterization and biological activities of exopolysaccharides from a new cold-adapted yeast: *Rhodotorula mucilaginosa* sp. GUMS16. Int. J. Biol. Macromol. 151, 268–277. doi: 10.1016/j.ijbiomac.2020.02.20, 32087227

[ref25] Hassan M. A. Hozien S. T. Abdel Wahab M. M. Hassan A. M. (2022). Ameliorative effect of selenium yeast supplementation on the physio-pathological impacts of chronic exposure to glyphosate and or malathion in *Oreochromis niloticus*. BMC Vet. Res. 18:159. doi: 10.1186/s12917-022-03261-0, 35501865 PMC9063350

[ref26] Hassan F. U. Liu C. Mehboob M. Bilal R. M. Arain M. A. Siddique F. . (2023). Potential of dietary hemp and cannabinoids to modulate immune response to enhance health and performance in animals: opportunities and challenges. Front. Immunol. 14:1285052. doi: 10.3389/fimmu.2023.1285052, 38111585 PMC10726122

[ref27] Honarpisheh P. Bryan R. M. McCullough L. D. (2022). Aging microbiota-gut-brain Axis in stroke risk and outcome. Circ. Res. 130, 1112–1144. doi: 10.1161/circresaha.122.319983, 35420913 PMC9674376

[ref28] Huang K. Bai H. Meng C. Kashif M. Wei Z. Tang Z. . (2025). Deciphering the ammonia transformation mechanism of a novel marine multi-stress-tolerant yeast, Pichia kudriavzevii HJ2, as revealed by integrated omics analysis. Appl. Environ. Microbiol. 91:e0221124. doi: 10.1128/aem.02211-24, 40338088 PMC12175507

[ref29] Ikram M. Javed B. Raja N. I. Mashwani Z. U. (2021). Biomedical potential of plant-based selenium nanoparticles: a comprehensive review on therapeutic and mechanistic aspects. Int. J. Nanomedicine 16, 249–268. doi: 10.2147/ijn.S295053, 33469285 PMC7811472

[ref30] Jing J. Zeng H. Shao Q. Tang J. Wang L. Jia G. . (2023). Selenomethionine alleviates environmental heat stress induced hepatic lipid accumulation and glycogen infiltration of broilers via maintaining mitochondrial and endoplasmic reticulum homeostasis. Redox Biol. 67:102912. doi: 10.1016/j.redox.2023.102912, 37797371 PMC10622879

[ref31] Jomova K. Alomar S. Y. Alwasel S. H. Nepovimova E. Kuca K. Valko M. (2024). Several lines of antioxidant defense against oxidative stress: antioxidant enzymes, nanomaterials with multiple enzyme-mimicking activities, and low-molecular-weight antioxidants. Arch. Toxicol. 98, 1323–1367. doi: 10.1007/s00204-024-03696-4, 38483584 PMC11303474

[ref32] Ju H. Chen S. Xue Y. Zhang X. Wang Y. (2021). The role of Nrf2 pathway in alleviating fluorine-induced apoptosis by different selenium sources in the chicken duodenum and jejunum. Ecotoxicol. Environ. Saf. 224:112708. doi: 10.1016/j.ecoenv.2021.112708, 34461318

[ref33] Kang K. Deng X. Xie W. Chen J. Lin H. Chen Z. (2023). *Rhodotorula mucilaginosa* ZTHY2 attenuates cyclophosphamide-induced immunosuppression in mice. Animals (Basel) 13:3376. doi: 10.3390/ani13213376, 37958131 PMC10648412

[ref34] Kayrouz C. M. Huang J. Hauser N. Seyedsayamdost M. R. (2022). Biosynthesis of selenium-containing small molecules in diverse microorganisms. Nature 610, 199–204. doi: 10.1038/s41586-022-05174-2 36071162

[ref35] Kieliszek M. Błażejak S. (2013). Selenium: significance, and outlook for supplementation. Nutrition 29, 713–718. doi: 10.1016/j.nut.2012.11.012, 23422539

[ref36] Kieliszek M. Kot A. M. Kolotylo V. (2023). Bioaccumulation of selenium and production of carotenoids by the yeast *Rhodotorula mucilaginosa*. Biocatal. Agric. Biotechnol. 53:102903. doi: 10.1016/j.bcab.2023.102903

[ref37] Kim S. J. Miller B. Kumagai H. Silverstein A. R. Flores M. Yen K. (2021). Mitochondrial-derived peptides in aging and age-related diseases. Geroscience 43, 1113–1121. doi: 10.1007/s11357-020-00262-5, 32910336 PMC8190245

[ref38] Kundu P. Lee H. U. Garcia-Perez I. Tay E. X. Y. Kim H. Faylon L. E. . (2019). Neurogenesis and prolongevity signaling in young germ-free mice transplanted with the gut microbiota of old mice. Sci. Transl. Med. 11:eaau4760. doi: 10.1126/scitranslmed.aau4760, 31723038

[ref39] Lawton K. A. Berger A. Mitchell M. Milgram K. E. Evans A. M. Guo L. . (2008). Analysis of the adult human plasma metabolome. Pharmacogenomics 9, 383–397. doi: 10.2217/14622416.9.4.383, 18384253

[ref40] Li H. Huang L. Zhang Y. Yan Y. (2020). Production, characterization and immunomodulatory activity of an extracellular polysaccharide from *Rhodotorula mucilaginosa* YL-1 isolated from sea salt field. Mar. Drugs 18:595. doi: 10.3390/md18120595, 33256151 PMC7760879

[ref41] Li X. Li C. Zhang W. Wang Y. Qian P. Huang H. (2023). Inflammation and aging: signaling pathways and intervention therapies. Signal Transduct. Target. Ther. 8:239. doi: 10.1038/s41392-023-01502-8, 37291105 PMC10248351

[ref42] Liang J. Wang Y. Wang T. Chu C. Yi J. Liu Z. (2025). Enhancing fermented vegetable flavor with *Lactobacillus plantarum* and *Rhodotorula mucilaginosa*. Food Res. Int. 200:115500. doi: 10.1016/j.foodres.2024.115500, 39779143

[ref43] Liu S. Wang R. Zhang Y. Dai Y. Zhang S. Lin X. . (2025). Effect of co-fermentation of *Rhodotorula mucilaginosa* with *Saccharomyces cerevisiae* on the quality and flavor profile of cider. Food Biosci. 64:105973. doi: 10.1016/j.fbio.2025.105973

[ref44] Loh J. S. Mak W. Q. Tan L. K. S. Ng C. X. Chan H. H. Yeow S. H. . (2024). Microbiota-gut-brain axis and its therapeutic applications in neurodegenerative diseases. Signal Transduct. Target. Ther. 9:37. doi: 10.1038/s41392-024-01743-1, 38360862 PMC10869798

[ref45] Ma X. Zhou B. Jiang L. Xie M. Rong Z. Yin S. . (2025). Microbial interactions between *Lactoplantibacillus plantarum* and *Rhodotorula mucilaginosa* in the fermented fish juice system. Food Res. Int. 208:116166. doi: 10.1016/j.foodres.2025.116166, 40263786

[ref46] Maldonado E. Morales-Pison S. Urbina F. Solari A. (2023). Aging hallmarks and the role of oxidative stress. Antioxidants (Basel) 12:651. doi: 10.3390/antiox12030651, 36978899 PMC10044767

[ref47] Mann E. R. Lam Y. K. Uhlig H. H. (2024). Short-chain fatty acids: linking diet, the microbiome and immunity. Nat. Rev. Immunol. 24, 577–595. doi: 10.1038/s41577-024-01014-8, 38565643

[ref48] Miao X. Niu H. Sun M. Dong X. Hua M. Su Y. . (2024). A comparative study on the nutritional composition, protein structure and effects on gut microbiota of 5 fermented soybean products (FSPs). Food Res. Int. 183:114199. doi: 10.1016/j.foodres.2024.114199, 38760132

[ref49] Militello R. Luti S. Gamberi T. Pellegrino A. Modesti A. Modesti P. A. (2024). Physical activity and oxidative stress in aging. Antioxidants (Basel) 13:557. doi: 10.3390/antiox13050557, 38790662 PMC11117672

[ref50] Newman R. Waterland N. Moon Y. Tou J. C. (2019). Selenium biofortification of agricultural crops and effects on plant nutrients and bioactive compounds important for human health and disease prevention - a review. Plant Foods Hum. Nutr. 74, 449–460. doi: 10.1007/s11130-019-00769-z, 31522406

[ref51] Nie X. Yang X. He J. Liu P. Shi H. Wang T. . (2023). Bioconversion of inorganic selenium to less toxic selenium forms by microbes: a review. Front. Bioeng. Biotechnol. 11:1167123. doi: 10.3389/fbioe.2023.1167123, 36994362 PMC10042385

[ref52] Pabst O. Hornef M. W. Schaap F. G. Cerovic V. Clavel T. Bruns T. (2023). Gut-liver axis: barriers and functional circuits. Nat. Rev. Gastroenterol. Hepatol. 20, 447–461. doi: 10.1038/s41575-023-00771-6, 37085614

[ref53] Pan R. Y. Zhang J. Wang J. Wang Y. Li Z. Liao Y. . (2022). Intermittent fasting protects against Alzheimer's disease in mice by altering metabolism through remodeling of the gut microbiota. Nat. Aging 2, 1024–1039. doi: 10.1038/s43587-022-00311-y, 37118092

[ref54] Panyard D. J. Yu B. Snyder M. P. (2022). The metabolomics of human aging: advances, challenges, and opportunities. Sci. Adv. 8:eadd6155. doi: 10.1126/sciadv.add6155, 36260671 PMC9581477

[ref55] Rong Y. Gao J. Kuang T. Chen J. Li J. A. Huang Y. . (2021). DIAPH3 promotes pancreatic cancer progression by activating selenoprotein TrxR1-mediated antioxidant effects. J. Cell. Mol. Med. 25, 2163–2175. doi: 10.1111/jcmm.16196, 33345387 PMC7882936

[ref56] Rua R. M. Nogales F. Carreras O. Ojeda M. L. (2023). Selenium, selenoproteins and cancer of the thyroid. J. Trace Elem. Med. Biol. 76:127115. doi: 10.1016/j.jtemb.2022.127115, 36481604

[ref57] Rudnicka E. Napierała P. Podfigurna A. Męczekalski B. Smolarczyk R. Grymowicz M. (2020). The World Health Organization (WHO) approach to healthy ageing. Maturitas 139, 6–11. doi: 10.1016/j.maturitas.2020.05.018, 32747042 PMC7250103

[ref58] Schaum N. Lehallier B. Hahn O. Pálovics R. Hosseinzadeh S. Lee S. E. . (2020). Ageing hallmarks exhibit organ-specific temporal signatures. Nature 583, 596–602. doi: 10.1038/s41586-020-2499-y, 32669715 PMC7757734

[ref59] Staneviciene I. Sulinskiene J. Sadauskiene I. Liekis A. Ruzgaite A. Naginiene R. . (2022). Effect of selenium on the Iron homeostasis and oxidative damage in brain and liver of mice. Antioxidants (Basel) 11:1216. doi: 10.3390/antiox11071216, 35883707 PMC9311717

[ref60] Su M. Tang T. Tang W. Long Y. Wang L. Liu M. (2023). Astragalus improves intestinal barrier function and immunity by acting on intestinal microbiota to treat T2DM: a research review. Front. Immunol. 14:1243834. doi: 10.3389/fimmu.2023.1243834, 37638043 PMC10450032

[ref61] Sumana S. L. Chen H. Shui Y. Zhang C. Yu F. Zhu J. . (2023). Effect of dietary selenium on the growth and immune systems of fish. Animals (Basel) 13:2978. doi: 10.3390/ani13182978, 37760378 PMC10525757

[ref62] Sun L. Liu G. Xu D. Wu Z. Ma L. Victoria S. M. . (2021). Milk selenium content and speciation in response to supranutritional selenium yeast supplementation in cows. Anim. Nutr. 7, 1087–1094. doi: 10.1016/j.aninu.2021.07.006, 34738039 PMC8545651

[ref63] Tian H. Zhang B. Di J. Jiang G. Chen F. Li H. . (2012). Keap1: one stone kills three birds Nrf2, IKKβ and Bcl-2/Bcl-xL. Cancer Lett. 325, 26–34. doi: 10.1016/j.canlet.2012.06.007 22743616

[ref64] Tilg H. Adolph T. E. Trauner M. (2022). Gut-liver axis: pathophysiological concepts and clinical implications. Cell Metab. 34, 1700–1718. doi: 10.1016/j.cmet.2022.09.017, 36208625

[ref66] Wang W. Liu F. Xu C. Liu Z. Ma J. Gu L. . (2021). *Lactobacillus plantarum* 69-2 combined with Galacto-oligosaccharides alleviates d-galactose-induced aging by regulating the AMPK/SIRT1 signaling pathway and gut microbiota in mice. J. Agric. Food Chem. 69, 2745–2757. doi: 10.1021/acs.jafc.0c06730, 33565862

[ref67] Wang Y. Wang P. Yuan S. Du X. Yan R. Wang X. . (2025). Reversal of BCAA-driven inflammatory senescence by traditional herbal oil prevents atopic dermatitis relapse. Phytomed. Int. J. Phytother. Phytopharm. 148:157425. doi: 10.1016/j.phymed.2025.157425, 41161187

[ref68] Wang R. Zeng Y. Liang J. Zhang H. Yi J. Liu Z. (2024). Effect of *Rhodotorula mucilaginosa* inoculation on the aroma development of a fermented vegetables simulated system. Food Res. Int. 179:113941. doi: 10.1016/j.foodres.2024.113941, 38342554

[ref69] Wu J. Hu Y. Zhao N. Yang W. Chen Z. (2025). The active roles of *Rhodotorula mucilaginosa* ZTHY2 in regulating antioxidant capacity and immune function of Leizhou black ducks. Front. Vet. Sci. 12:1494892. doi: 10.3389/fvets.2025.1494892, 39950088 PMC11821949

[ref70] Wu L. Xie X. Li Y. Liang T. Zhong H. Yang L. . (2022). Gut microbiota as an antioxidant system in centenarians associated with high antioxidant activities of gut-resident Lactobacillus. NPJ Biofilms and Microbiomes 8:102. doi: 10.1038/s41522-022-00366-0, 36564415 PMC9789086

[ref71] Xu Z. Hu J. Zhang Y. Bai L. (2023). Evaluation of largemouth bass (*Micropterus salmoide*) fed selenium yeast diets: growth, histopathology, antioxidant ability, and apoptosis. Aquacult. Rep. 29:101505. doi: 10.1016/j.aqrep.2023.101505

[ref72] Xue C. Li G. Zheng Q. Gu X. Shi Q. Su Y. . (2023). Tryptophan metabolism in health and disease. Cell Metab. 35, 1304–1326. doi: 10.1016/j.cmet.2023.06.004, 37352864

[ref73] Zhai Q. Cen S. Li P. Tian F. Zhao J. Zhang H. . (2018). Effects of dietary selenium supplementation on intestinal barrier and immune responses associated with its modulation of gut microbiota. Environ. Sci. Technol. Lett. 5, 724–730. doi: 10.1021/acs.estlett.8b00563

[ref74] Zhang Z. Mu X. Cao Q. Shi Y. Hu X. Zheng H. (2022). Honeybee gut *Lactobacillus* modulates host learning and memory behaviors via regulating tryptophan metabolism. Nat. Commun. 13:2037. doi: 10.1038/s41467-022-29760-0, 35440638 PMC9018956

[ref75] Zhang Z. H. Wen L. Wu Q. Y. Chen C. Zheng R. Liu Q. . (2017). Long-term dietary supplementation with selenium-enriched yeast improves cognitive impairment, reverses synaptic deficits, and mitigates tau pathology in a triple transgenic mouse model of Alzheimer's disease. J. Agric. Food Chem. 65, 4970–4979. doi: 10.1021/acs.jafc.7b01465, 28578584

[ref76] Zhang Z. H. Wu Q. Y. Chen C. Zheng R. Chen Y. Ni J. Z. . (2018). Comparison of the effects of selenomethionine and selenium-enriched yeast in the triple-transgenic mouse model of Alzheimer's disease. Food Funct. 9, 3965–3973. doi: 10.1039/c7fo02063e, 29974078

[ref77] Zhang S. Wu Z. Zhang S. Ru Y. Wang Q. Tong H. . (2025). The intricate microbial-gut-brain axis in Alzheimer's disease: a review of microbiota-targeted strategies. Food Funct. 16, 8320–8344. doi: 10.1039/d5fo03139g, 41085348

[ref78] Zhao L. Yang H. Xu M. Xin W. Wang C. Lian Y. . (2019). Stevia residue extract ameliorates oxidative stress in d-galactose-induced aging mice via Akt/Nrf2/HO-1 pathway. J. Funct. Foods 52, 587–595. doi: 10.1016/j.jff.2018.11.044.

