# Supplementary material for: The selenium-enriched Rhodotorula mucilaginosa JAASRY1 improved oxidative stress during the aging process via the gut-liver-brain axis
Source: Front Microbiol. 2026 Jun 2;17:1809542. doi: 10.3389/fmicb.2026.1809542 (PMC13269060; doi:10.3389/fmicb.2026.1809542)
Supplement: Supplementary file 1 [file Table_1.docx]

Supplementary Material

# Supplementary Figures and Tables


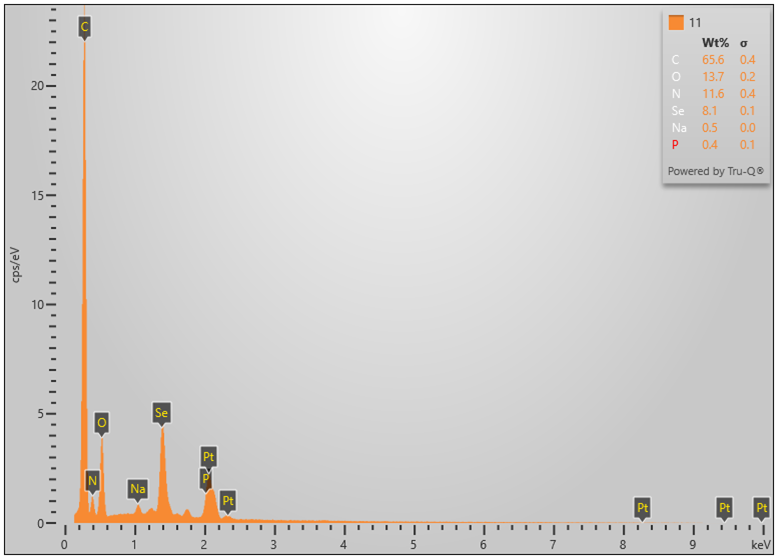


**Supplementary Figure 1.** Energy spectrum of *Se-RMSRY*.


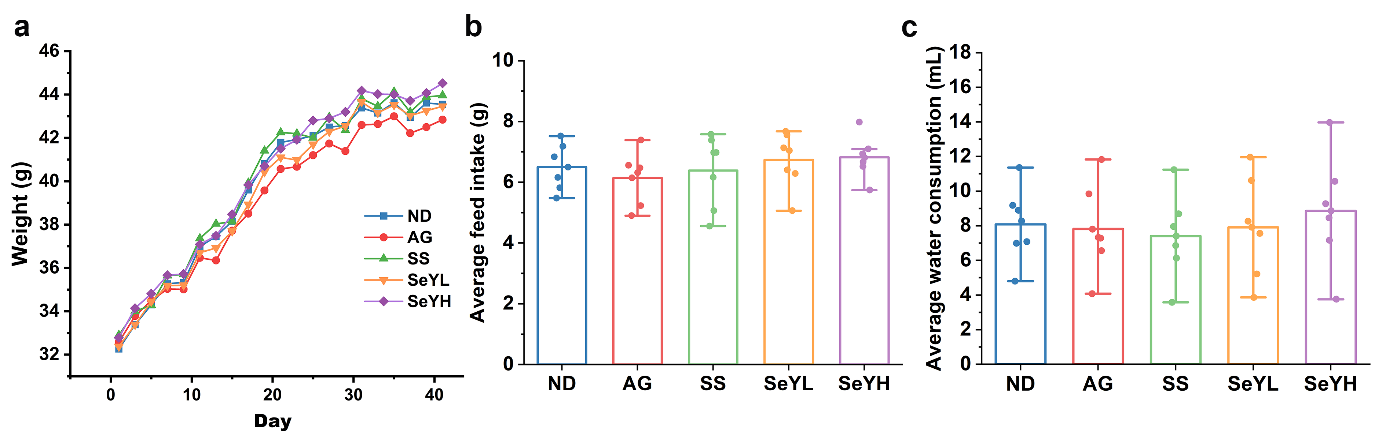
**Supplementary Figure 2.** Body weight gain (a) and changes in diet (b) and water intake (c) in mice.
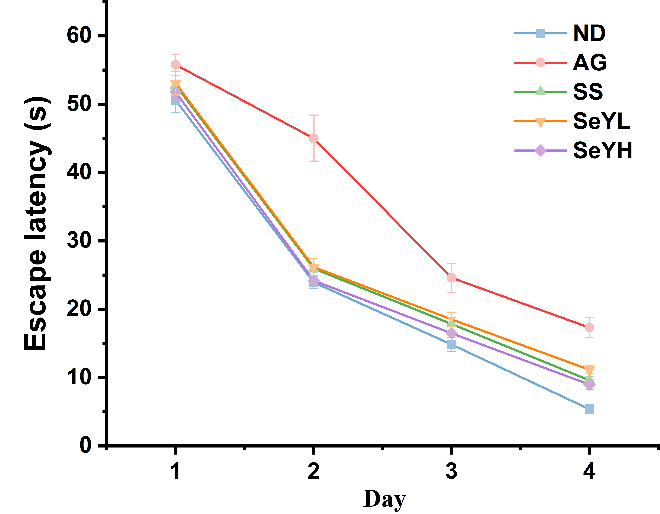

**Supplementary Figure 3.** Escape latency changes in the spatial acquisition trials.


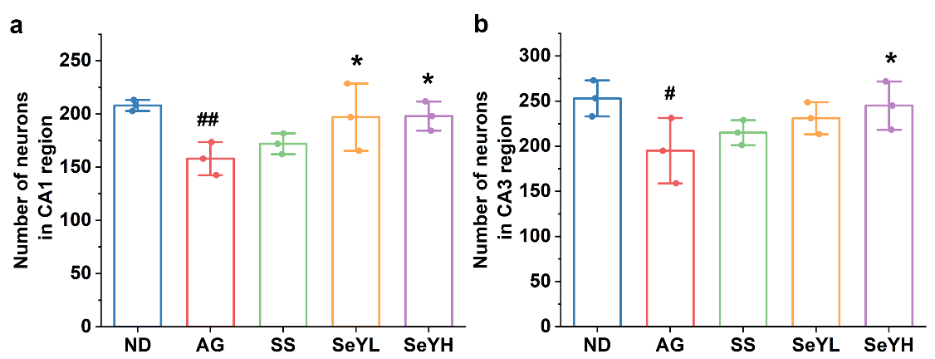


**Supplementary Figure 4.** Number of CA1 (a) and CA3 (b) neurons in mice hippocampus.


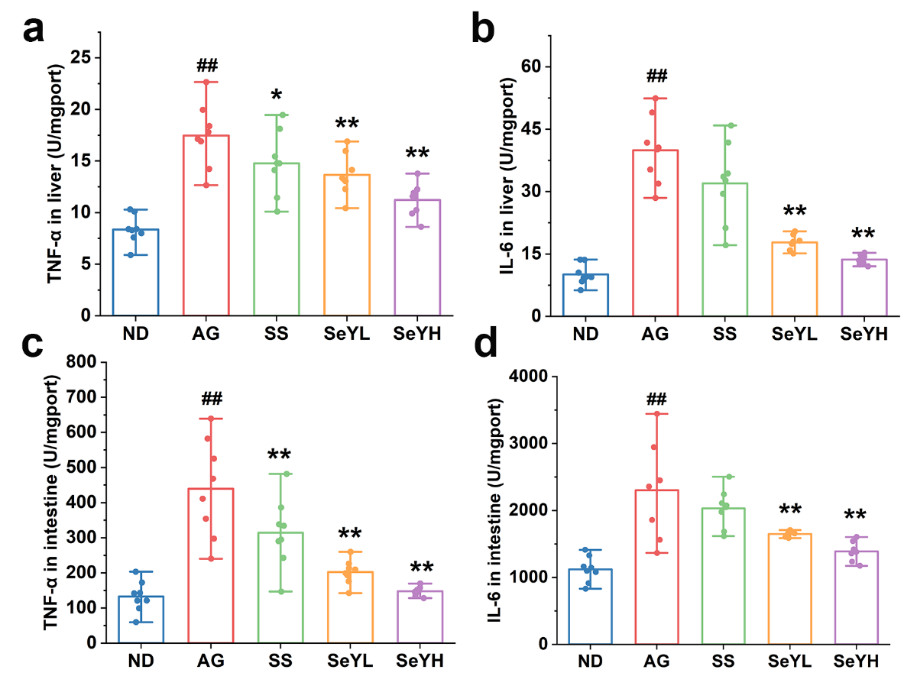


**Supplementary Figure 5.** Changes in inflammatory cytokines in the liver and jejunum of mice. (a) TNF-α in the liver, (b) IL-6 in the liver, (c) TNF-α in the jejunum, and (d) IL-6 in the jejunum. The ‘#’ represented a significant change compared to the ND group, *p* < 0.05. The ‘##’ represented a highly significant change compared to the ND group, *p* < 0.01. The ‘*’ represented a significant change compared to the AG group, *p* < 0.05. The ‘**’ represented a highly significant change compared to the AG group, *p* < 0.01.


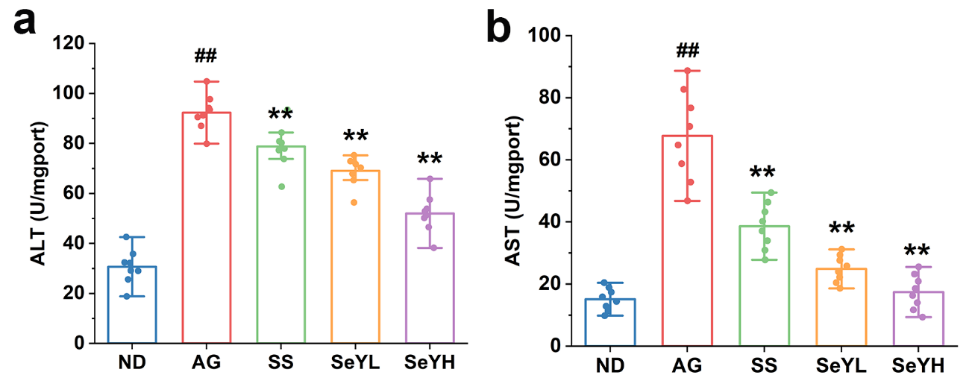


**Supplementary Figure 6.** Evaluation of liver function in mice. (a) ALT, (b) AST. The ‘#’ represented a significant change compared to the ND group, *p* < 0.05. The ‘##’ represented a highly significant change compared to the ND group, *p* < 0.01. The ‘*’ represented a significant change compared to the AG group, *p* < 0.05. The ‘**’ represented a highly significant change compared to the AG group, *p* < 0.01.


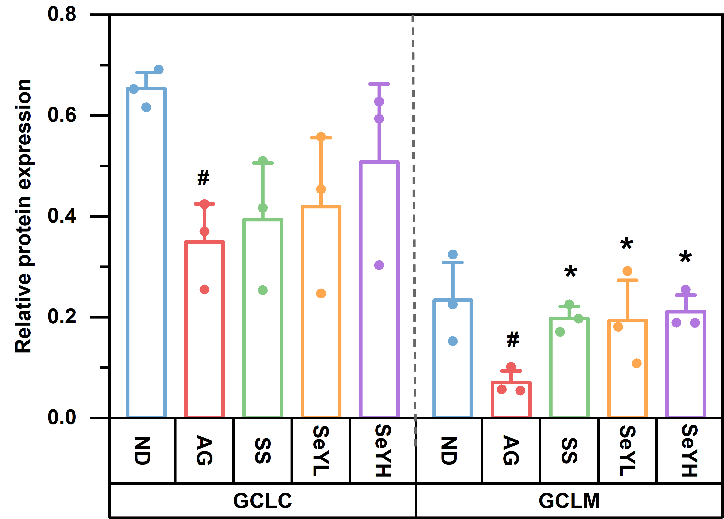


**Supplementary Figure 7.** Relative proteins expression of GCLC and GCLM. The ‘#’ represented a significant change compared to the ND group, *p* < 0.05. The ‘##’ represented a highly significant change compared to the ND group, *p* < 0.01. The ‘*’ represented a significant change compared to the AG group, *p* < 0.05. The ‘**’ represented a highly significant change compared to the AG group, *p* < 0.01


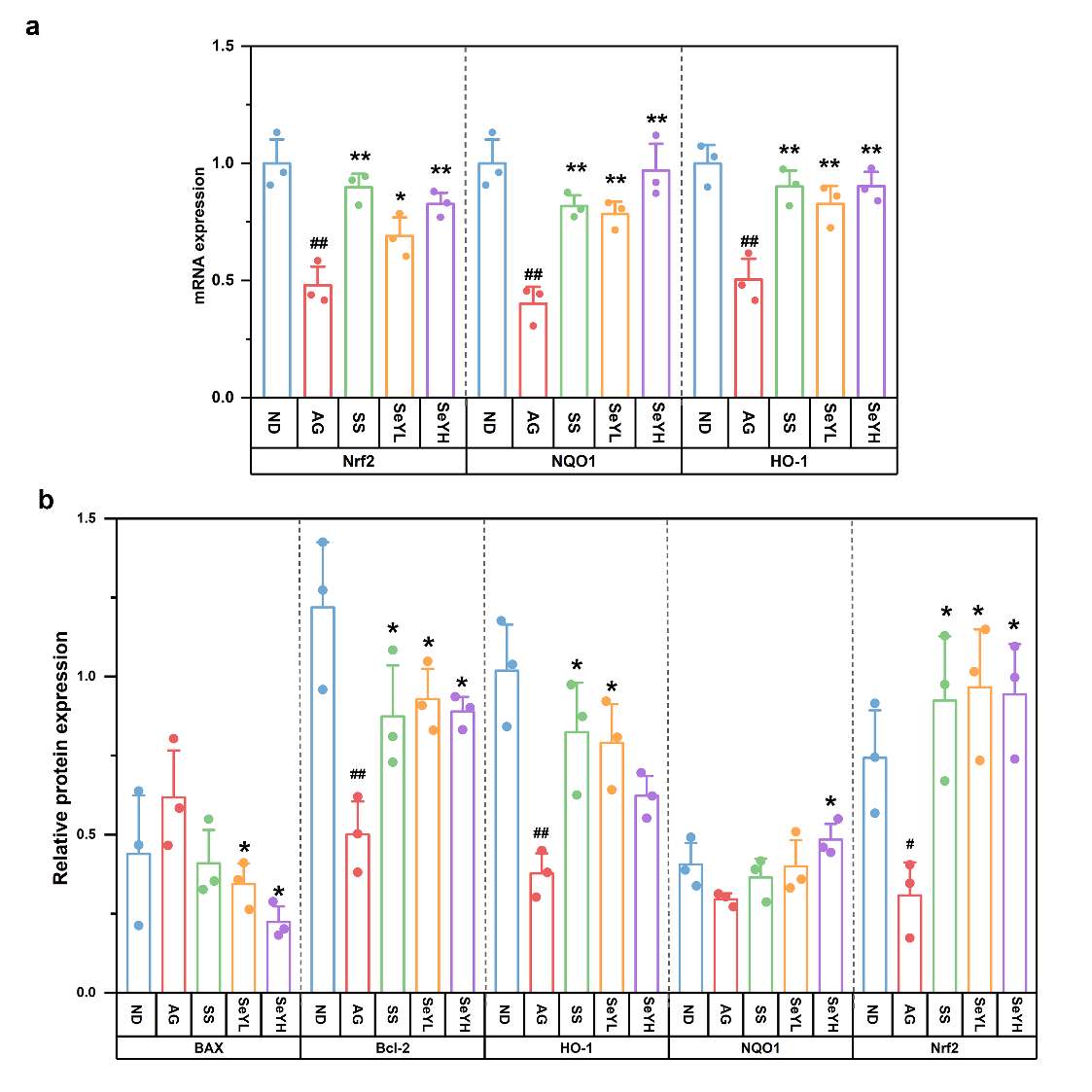


**Supplementary Figure 8.** mRNA expression (a) and relative protein expression (b) in intestine. The ‘#’ represented a significant change compared to the ND group, *p* < 0.05. The ‘##’ represented a highly significant change compared to the ND group, *p* < 0.01. The ‘*’ represented a significant change compared to the AG group, *p* < 0.05. The ‘**’ represented a highly significant change compared to the AG group, *p* < 0.01.

**Supplementary Table 1.** Changes in body weight of mice

Table S1 Changes in body weight of mice

| Group | Initial weigh (g) | Final weight (g) | Weight gain rate |
| --- | --- | --- | --- |
| ND | 32.26±1.3 | 43.55±2.06 | 31.12% |
| AG | 32.58±1.26 | 42.84±3.48 | 28.99% |
| SS | 32.9±2.12 | 43.96±3.81 | 33.62% |
| SeYL | 32.37±1.87 | 43.46±3.28 | 30.43% |
| SeYH | 32.78±1.76 | 44.52±2.74 | 35.81% |
